# Serial Femtosecond Crystallography Reveals that Photoactivation in a Fluorescent Protein Proceeds via the Hula Twist Mechanism

**DOI:** 10.1021/jacs.3c02313

**Published:** 2023-07-07

**Authors:** Alisia Fadini, Christopher D.M. Hutchison, Dmitry Morozov, Jeffrey Chang, Karim Maghlaoui, Samuel Perrett, Fangjia Luo, Jeslyn C.X. Kho, Matthew G. Romei, R. Marc L. Morgan, Christian M. Orr, Violeta Cordon-Preciado, Takaaki Fujiwara, Nipawan Nuemket, Takehiko Tosha, Rie Tanaka, Shigeki Owada, Kensuke Tono, So Iwata, Steven G. Boxer, Gerrit Groenhof, Eriko Nango, Jasper J. van Thor

**Affiliations:** † Department of Life Sciences, Faculty of Natural Sciences, Imperial College London, London SW7 2AZ, U.K.; ‡ Nanoscience Center and Department of Chemistry, University of Jyväskylä, Jyväskylä 40014, Finland; § Department of Physics, Stanford University, Stanford, California 94305, United States; ∥ RIKEN Spring-8 Center, 1-1-1 Kouto, Sayo, Sayo, Hyogo 679-5148, Japan; ⊥ Department of Chemistry, Stanford University, Stanford, California 94305, United States; # Diamond Light Source Ltd, Harwell Science & Innovation Campus, Didcot OX11 0DE, U.K.; ¶ Institute of Multidisciplinary Research for Advanced Materials, Tohoku University, 2-1-1 Katahira, Aoba, Sendai, Miyagi 980-8577, Japan; ∇ Department of Cell Biology, Graduate School of Medicine, Kyoto University, Yoshidakonoe, Sakyo, Kyoto 606-8501, Japan; ○ Japan Synchrotron Radiation Research Institute, 1-1-1 Kouto, Sayo, Sayo, Hyogo 679-5198, Japan

## Abstract

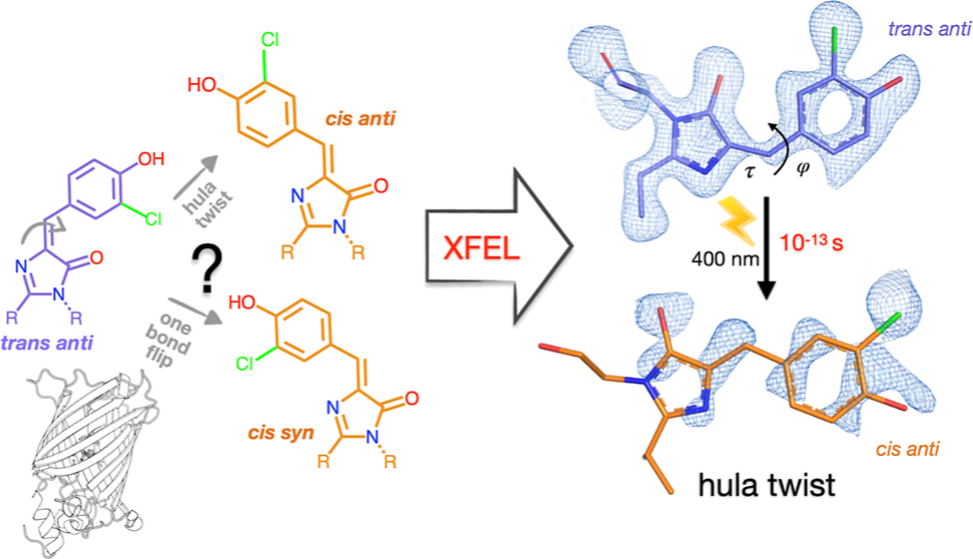

Chromophore *cis/trans* photoisomerization is a fundamental process in chemistry and in the activation of many photosensitive proteins. A major task is understanding the effect of the protein environment on the efficiency and direction of this reaction compared to what is observed in the gas and solution phases. In this study, we set out to visualize the hula twist (HT) mechanism in a fluorescent protein, which is hypothesized to be the preferred mechanism in a spatially constrained binding pocket. We use a chlorine substituent to break the twofold symmetry of the embedded phenolic group of the chromophore and unambiguously identify the HT primary photoproduct. Through serial femtosecond crystallography, we then track the photoreaction from femtoseconds to the microsecond regime. We observe signals for the photoisomerization of the chromophore as early as 300 fs, obtaining the first experimental structural evidence of the HT mechanism in a protein on its femtosecond-to-picosecond timescale. We are then able to follow how chromophore isomerization and twisting lead to secondary structure rearrangements of the protein β-barrel across the time window of our measurements.

## Introduction

The light-induced *cis/trans* isomerization of a chromophore double bond is a key reaction in photochemistry. In the dynamical response that follows photon absorption, photoisomerization has been shown to be the primary event for a variety of photoreceptors, such as visual pigments, and for the toolbox of photoactivatable proteins used for super-resolution microscopy.^1−4^ A detailed understanding of the reaction pathway and of how it is steered by the protein environment is key for the rational design of more effective photosystems to employ in nanoscopy,^5−8^ optogenetics,^2,9−13^ and fluorescence biosensing.^14,15^

**Figure 1:**
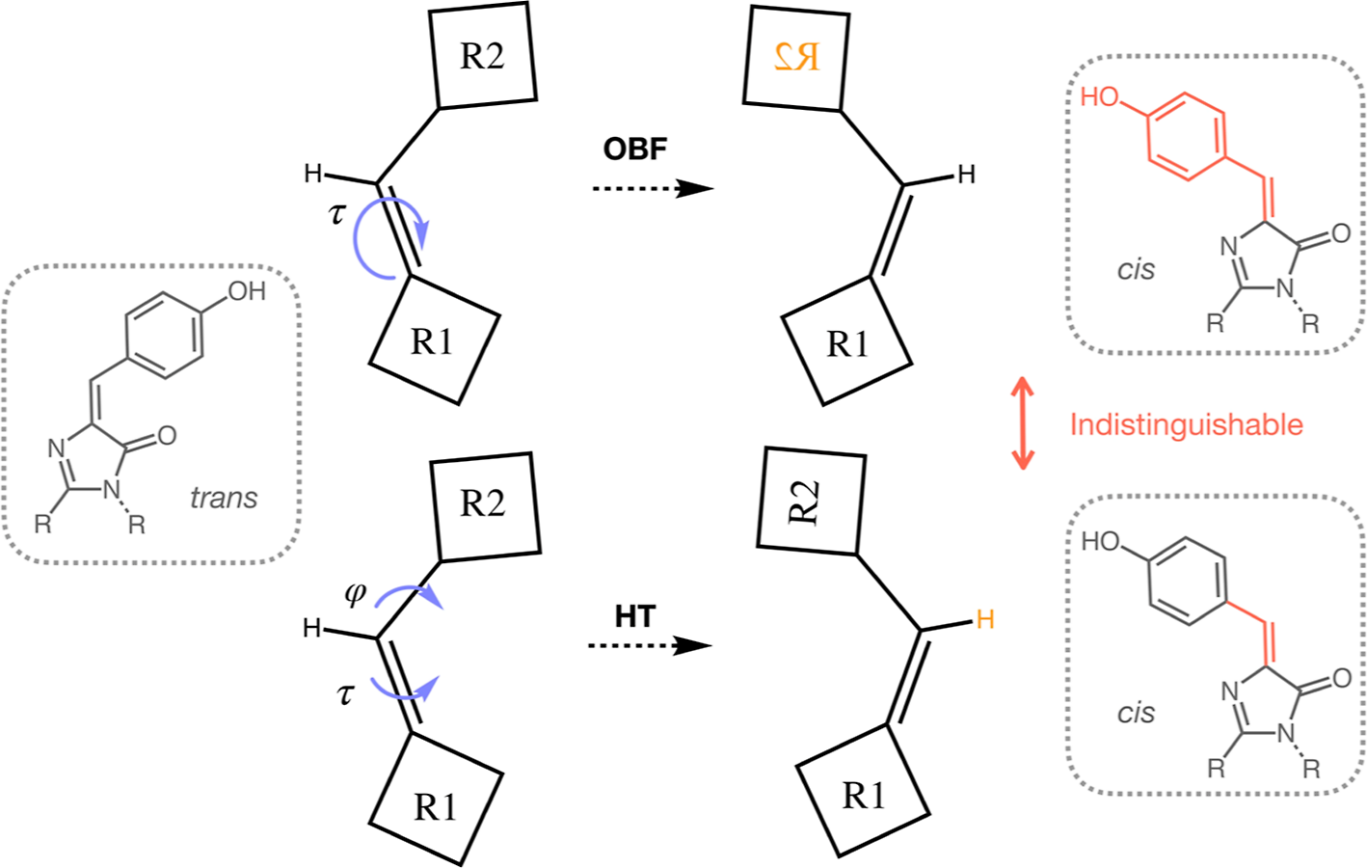
Schematic of potential chromophore photoisomerization pathways. Two possible pathways for the *cis/trans* photoisomerization of a chromophore are the OBF pathway or the HT pathway, both shown here schematically. The OBF mechanism involves the rotation of only the isomerizing bond, τ, and is expected to sweep a large volume, as half of the molecule is flipped in the process. In the HT pathway, on the other hand, both τ and its neighboring bond ϕ rotate simultaneously. The products formed by these two pathways are indistinguishable if the part of the molecule that flips is symmetric.

Despite the ubiquity of this reaction, the precise mechanism of photoisomerization in conjugated systems is hard to determine. Two conceivable pathways that a protein chromophore can follow for *cis/trans* photoisomerization are the one bond flip (OBF) and the hula twist (HT).^16^ In the conventional OBF, the only bond to rotate is the one undergoing isomerization (τ in Figure 1—top);^17^ half of the molecule needs to turn over, indicating that the OBF mechanism is expected to require a considerable amount of space to be available. In the 1980s, Liu and Asato reasoned that, in proteins, this volume-demanding transition seemed to be in contrast with the observed picosecond formation of an isomerized photoproduct: significant chromophore pocket residue rearrangements that might accompany a large volume sweep by the chromophore are unlikely to occur within such a timescale. As a volume-conserving alternative to the OBF for isomerization in bathorhodopsin, they proposed the HT mechanism, where both τ and the neighboring bond ϕ rotate simultaneously^18^ (Figure 1—bottom). Since it has become generally accepted that photoisomerization in rhodopsins occurs through a bicycle-pedal mechanism, in which the *cis* conformation is propagated along the chromophore by a concerted rotation about parallel pairs of double bonds and not through a HT.^19−21^ A recent time-resolved crystallographic study has in fact obtained structural evidence for an aborted bicycle-pedal mechanism in a bovine rhodopsin starting at 1 ps.^22,23^ Nonetheless, crystallographic data supporting the presence of two volume-conserving isomerization pathways, including the HT, were obtained for nanosecond intermediates of the photoactive yellow protein (PYP) photocycle.^24^

Conclusive experimental evidence or consensus for whether GFP-like chromophores that are functionally embedded in proteins undergo OBF or HT as the primary photoactivation pathway has not yet been obtained.^16,25−32^ Two reasons for this are, first, that the primary photoproducts are formed on the ultrafast timescale and are very short-lived, requiring picosecond, or preferably femtosecond, time resolution to be distinguished. Second, the ground-state species formed immediately after photoisomerization by OBF and HT are indistinguishable if the part of the molecule that flips is symmetric (Figure 1). In this work, we address both these issues to determine the photoisomerization pathway in a construct of the reversibly switchable fluorescent protein rsEGFP2.^33^

**Figure 2:**
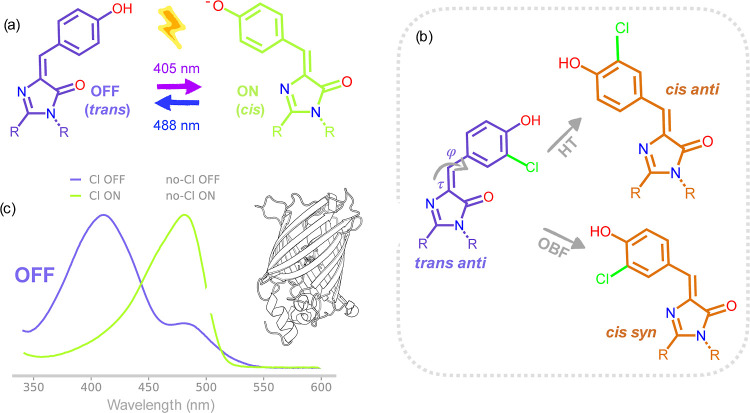
Cl-rsEGFP2 photoswitching. (a) Reversibly photoswitchable protein rsEGFP2 can be converted between a dark OFF state to a fluorescent ON state by specific frequencies of light. These changes are caused by a *trans*-to-*cis* isomerization and subsequent deprotonation of its embedded chromophore. (b) Addition of a chlorine substituent to the phenolate ring of the chromophore can break its C2 point group symmetry and allow us to distinguish between the HT and OBF mechanisms of chromophore photoisomerization in the first photoproduct of the OFF-to-ON reaction. The normalized absorption spectrum and structure of Cl-rsEGFP2 (c) possess very similar properties as the non-chlorinated protein: the OFF state absorbing predominantly at 400 nm and the ON state at around 480 nm, while the protein tertiary structure exhibits the β-barrel fold typical of GFP-like constructs. A 2 nm shift can be observed in the absorption profile between chlorinated and non-chlorinated constructs caused by the electron-withdrawing nature of chlorine.^47^

Reversibly switchable fluorescent proteins (RSFPs), such as rsEGFP2, can typically be converted between a fluorescent ON state and a non-fluorescent OFF state using specific photon wavelengths.^34,35^ They have been widely employed in super-resolution microscopy and imaging;^36−42^ rsEGFP2 in particular is a β-barrel protein that can be switched from its equilibrium ON state to the non-fluorescent state by 488 nm light, whereas the OFF-to-ON transition is achieved by illumination at 405 nm. In the fluorescent ON state, the chromophore is found as the anionic *cis* isomer, while the neutral *trans* isomer is responsible for the non-fluorescent OFF state (Figure 2a).^33,43^ Time-resolved structural and spectroscopic data previously obtained for the OFF-to-ON reaction of rsEGFP2^44,45^ identified the formation of a twisted chromophore intermediate within a couple of picoseconds and the presence of a ground-state protonated *cis* conformer 10 ns after photoexcitation, attributing deprotonation to a later, ground-state process. An experimental structural insight on the fundamental isomerization pathway and on the involvement of the speculated chromophore HT, however, remains lacking. As with previous studies, we focus here on the OFF-to-ON reaction, which has a higher quantum yield than the ON-to-OFF reaction.^43,46^ The 4-hydroxybenzylidene-1,2-dimethylimidazolinone chromophore’s phenol ring in rsEGFP2 has a C_2_ point group symmetry, leading to equivalent *cis* products regardless of the photoisomerization pathway. We exploit the introduction of a chlorine atom substituent^47^ to break this symmetry and distinguish between the products formed via the OBF and HT pathways, as previously suggested.^48^ On the basis of IUPAC recommendations,^49−51^ starting from a *trans* chromophore with a substituent *anti* to the double-bonded imidazolinone nitrogen, HT leads to a *cis anti* product, while the configuration formed via OBF is *cis syn* (Figure 2b). The motivation behind incorporation of chlorine in rsEGFP2 is therefore to obtain a construct with photoswitching capabilities that resemble those of rsEGFP2^48^ but that can be investigated to confirm either HT or OBF. We note below that there are structural and spectroscopic differences between the chlorinated and unmodified constructs and the observation of the HT pathway in the chlorinated construct we consider here does necessarily imply such process in other constructs.

In a prior study,^48^ the same rsEGFP2 construct containing a monochlorinated chromophore (Cl-rsEGFP2) was used to study the ON-to-OFF reaction and compare protein structures before and after irradiation. Structures were obtained by rapidly cryocooling crystals after a specific irradiation period and subsequently collecting diffraction data using synchrotron X-ray radiation. This method relied on the assumption that the cryocooled irradiated structure reflected the chromophore configuration immediately after photoisomerization but was not able to account for thermal bond rotations, which can interconvert *anti* and *syn* species, or any change in chromophore configuration induced by the freezing process itself. Here, by performing a time-resolved serial femtosecond crystallography (TR-SFX) experiment with sub-picosecond resolution, we have been able to unequivocally identify the primary photoproduct of the OFF-to-ON photoisomerization reaction: the *trans anti* chromophore in the OFF state of Cl-rsEGFP2 is photoexcited with a 400 nm photon, and clear signals showing the formation of a *cis anti* photoproduct are reported starting at delays of 300 fs. This, to our knowledge, is the first direct experimental structural evidence that unequivocally supports HT chromophore photoisomerization in a protein on its ultrafast timescale, more than 30 years after it was first hypothesized.

## Results and Discussion

### OFF-State SFX Structure for Cl-rsEGFP2

**Figure 3:**
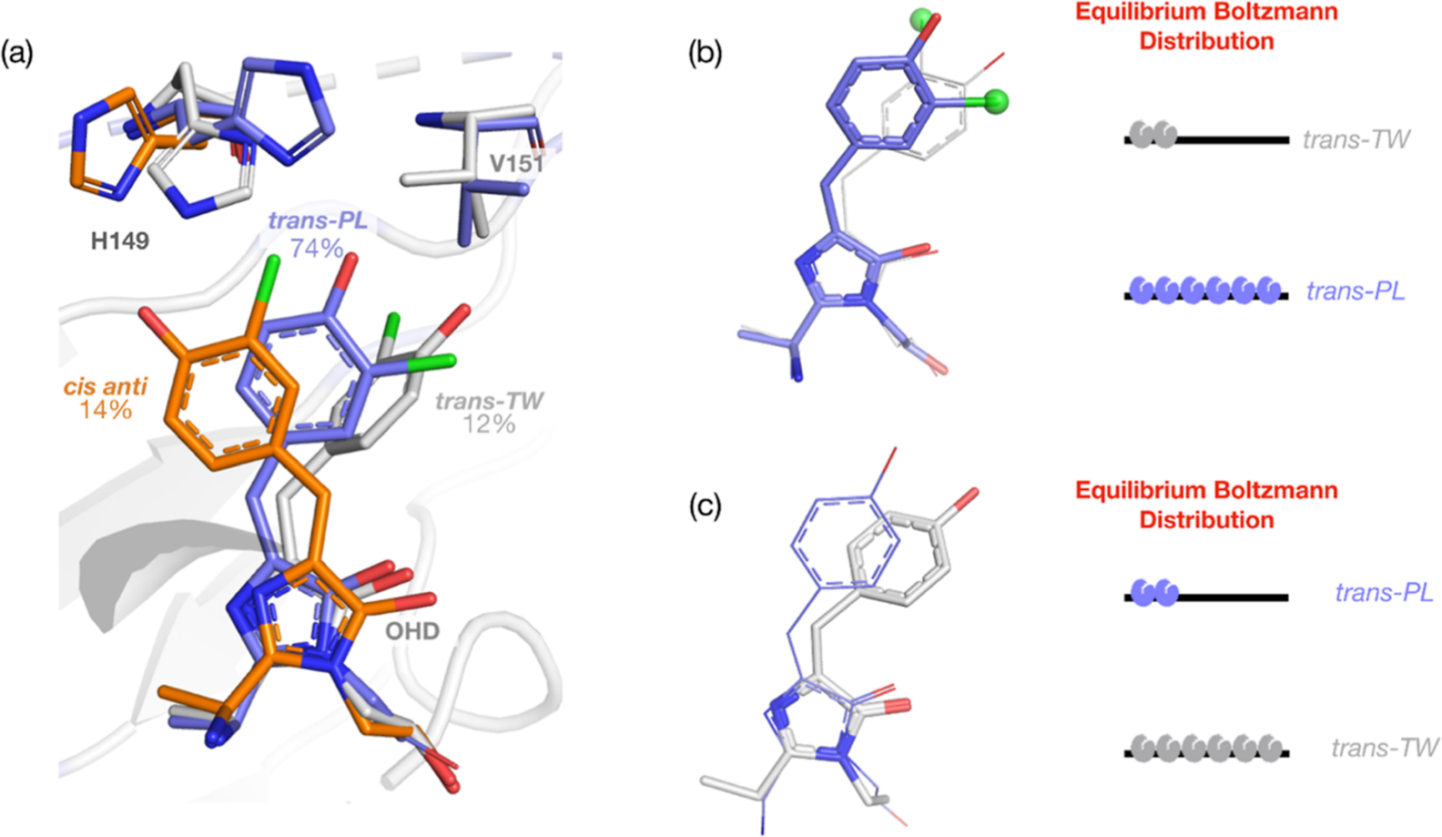
OFF-state chromophore configurations in Cl-rsEGFP2. (a) Refined Cl-rsEGFP2 dark OFF-state structure obtained from room-temperature serial femtosecond crystallography (PDB 8A6G). The predominant chromophore (OHD) conformation is the “planar” *trans anti* (*trans-PL*). Minor populations of a “twisted” *trans syn* (*trans-TW*) and of *cis anti* configurations are also modeled (refined occupancies of 12 and 14%, respectively). The three chromophore configurations are accompanied by three alternate His149 conformations. Two Val151 conformations are also resolved and matched to the *trans-PL* and *trans-TW* species. (b) OFF-state equilibrium *trans* structures of Cl-rsEGFP2 and (c) rsEGFP2.^44^ The major configuration found in the OFF-state chlorinated structure is *trans-PL*, while it is *trans-TW* when the chlorine substituent is not present. We presume that the heavy atom substitution results in a higher energy for the *trans-TW* state through increased steric hindrance. See also Figures S4–S6, Table S3, and Note S1.

Cl-rsEGFP2 maintains very similar switching properties to rsEGFP2 (Figures 2c and S1) and is expressed using amber suppression as described by Chang et al*.*^48^ Cl-rsEGFP2 microcrystals diffracted to 1.63 Å at the SACLA X-ray Free Electron Laser (XFEL) in Japan (Figure S2). Crystals were pre-illuminated with a 488 nm CW laser in order to photoaccumulate the OFF state. The OFF-to-ON transition was then initiated with a 75 fs 400 nm pump laser pulse and probed with the XFEL pulse at pump–probe delays between 300 fs and 1 μs. To obtain a ground-state room-temperature structure of the Cl-rsEGFP2 OFF state, dark data was collected in an interleaved manner (1:5 ratio) with the pump–probe data throughout our TR-SFX experiment. To account for pump laser scatter during sample delivery and to model a more accurate dark structure, a dataset was also collected with a negative pump delay (−5 ps). A dark structure (PDB 8A6G) was refined to the interleaved dark dataset (Figure 3a) and confirmed through 2mFo-DFc and mFo-DFc maps obtained with the −5 ps data (Figure S4). This presents the chromophore primarily in a planar *trans anti* configuration (named here *trans-PL*). Minor populations of a twisted *trans syn* (named *trans-TW*) and of a *cis anti* species were also manually refined to occupancies of 12 and 14%, respectively, as well as the accompanying alternate conformations of residues H149 and V151 (Figures 3 and S4). The *trans-PL* and *trans-TW* species we observe here have been previously reported in room-temperature crystal structures of rsEGFP2^44,52^ and in cryostructures of Cl-rsEGFP2^48^ (Table S3). Absorption spectra and quantum chemical calculations also point to the presence of these two forms in rsEGFP2 solutions.^52^ We expect that the ON-to-OFF reaction starting from the *cis* chromophore can therefore eventually result in either of these two *trans* conformations.

Adam et al.^52^ postulate a fast exchange between *trans-PL* and *trans-TW* in solution (on the order of ≈0.1 s) and propose that the two states are separated by a low energy barrier in the protein conformational energy landscape. In room-temperature rsEGFP2 SFX structures, the prevalent *trans* configuration is the twisted *trans-TW* (Figure S5 and ref (52)), suggesting that it is a lower energy state than *trans-PL*. In Cl-rsEGFP2, we observe the prevalent configuration to be *trans-PL* and presume that the transition from the planar to the twisted form is no longer energetically favorable due to the steric hindrance introduced by the bulk of the chlorine atom or to an electronic substituent effect (see also Note S1). This is also in line with the finding that an enlargement of a chromophore pocket residue (V151L) can shift the OFF state equilibrium almost completely to *trans-PL* in non-chlorinated rsEGFP2.^52^ The states observed in our dark OFF-state structure are shown in Figure 3b. For the scope of this time-resolved study, we expect that any observed photoinduced species result from the *trans-PL*-to-*cis* reaction; since the starting population of *trans-TW* in our dark-state structure is below 15%, we expect that any photoproduct resulting from photoexcitation of this state will not be detectable in our data.

### TR-SFX Data for Cl-rsEGFP2

#### Chromophore-Specific Changes

**Figure 4:**
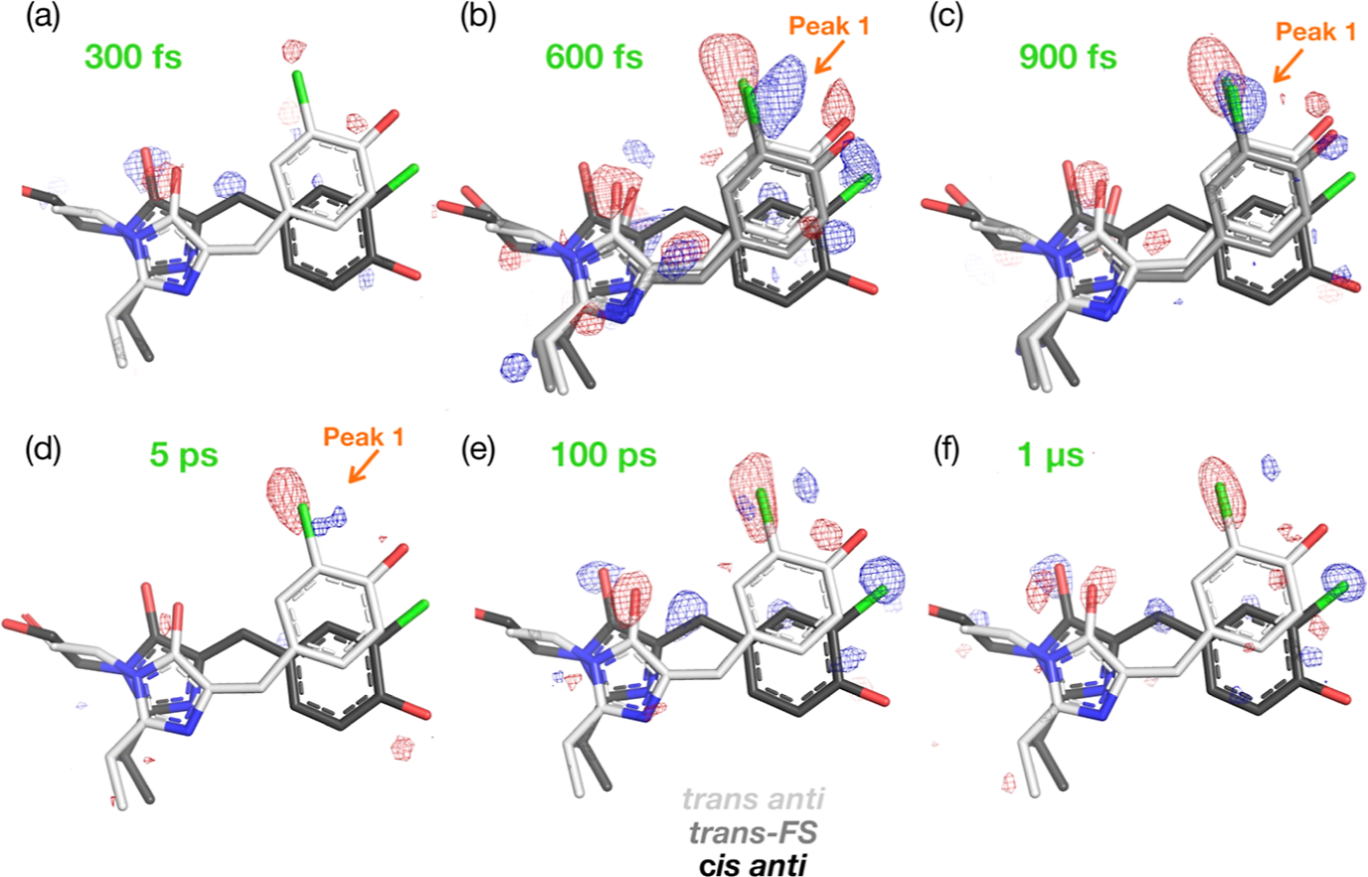
Time-resolved DED maps of Cl-rsEGFP2. *Q*-weighted DED maps for the collected pump–probe delays. Positive (blue) and negative (red) DED is shown at ±3.5 σ. The refined *trans anti* and *cis anti* species are shown in light and dark gray, respectively. In the 600 fs, 900 fs, and 5 ps maps, the feature “Peak 1″ is highlighted: this is the main indicator of the presence of a femtosecond intermediate, which we have called *trans-FS* and discussed with further details in the main text. PDBs (in order) 8A6N, 8A6O, 8A6P, 8A6Q, 8A6R, and 8A6S. See also Figures S7–S13.

Pump–probe TR-SFX data was collected for six delays: 300 fs, 600 fs, 900 fs, 5ps, 100 ps, and 1 μs. The pump laser power density used was 0.5 mJ/mm^2^. We generated *Q*-weighted difference electron density (DED) maps for these delays and improved signal-to-noise through principal component analysis using a python pipeline (Figures S7 and S8 and Supporting Information Procedures Section 1). The resulting maps are shown in Figure 4 at ±3.5σ. The initial negative density signals on the *trans-PL* imidazolinone ring oxygen are already visible at 300 fs and are accompanied by respective positive density on the same oxygen and on the *cis* methylene bridge. At 600 fs, there is a large negative density on the *trans-PL* Cl atom, and negative signals also on the two chromophore oxygens and methylene bridge. Positive density signals appear on both *cis anti* rings. In the 600 and 900 fs data, there is also a strong (+5σ) positive feature that we have labeled Peak 1 and suggests an upward shift of chlorine position (approximately 1 Å—Figure 4b,c). This peak is still weakly present in the 5 ps data (Figure 4d) and effectively absent in the 100 ps and 1 μs maps. We assign Peak 1 to an intermediate state, which we call *trans-FS* (Figure 4b), discussed in more detail below. At 100 ps and 1 μs, the *trans-PL* to *cis anti* features are very clear and are the prevalent signals at ±3.5σ: there are strong negative and positive features on the chromophore chlorine substituent and on the imidazolinone ring carbonyl, as well as positive density on the *cis anti* methylene bridge and the phenol ring (Figure 4e,f). These difference density signals thus point to increased population of the *cis anti* species and decreased population of *trans anti* starting from 300 fs, as well as possible formation of a femtosecond intermediate. In the analysis below, we investigate this further to refine the structure and occupancy of the *cis anti* and *trans-FS* conformations for each time point.

**Figure 5:**
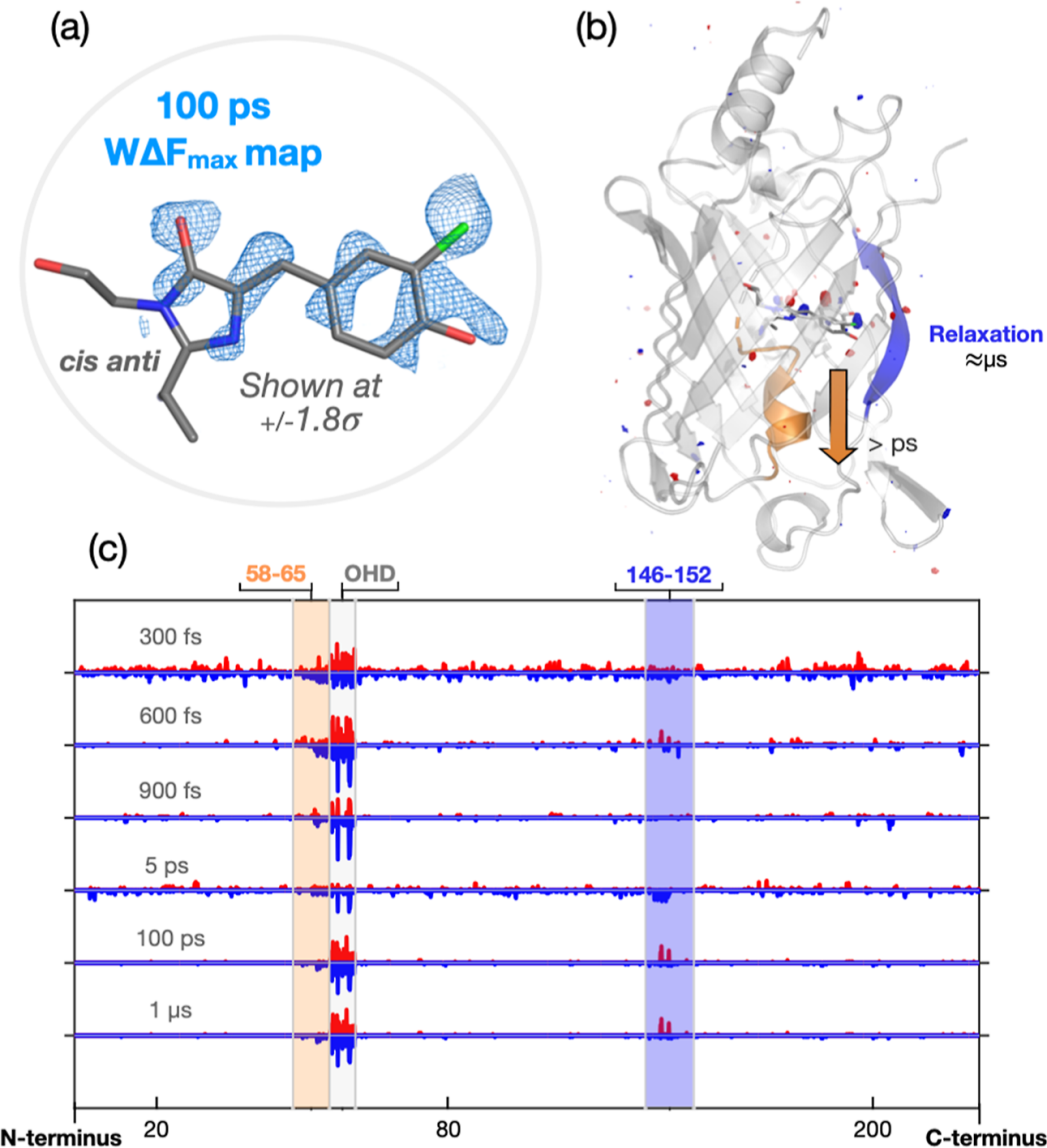
Light-induced changes across the entire Cl-rsEGFP2 protein structure. (a) The background-subtracted map (*W*Δ*F*_max_) for the 100 ps dataset clearly outlines the presence of the *cis anti* photoproduct. (b,c) The strongest signals in the *Q*-weighted DED maps for the collected time points are concentrated on the chromophore (OHD). However, by moving a spherical volume through all the atoms in the protein and integrating the negative and positive electron density within it, two other regions of variation stand out: the central α-helix (residues 58–67, orange) and the β-strand 7 (residues 146–152, blue). Signals in the α-helix are strongest in the early data points (c) and suggest a downward shift of the helix [(b) and Figure S15]. Negative density signals on β-strand 7 (Figure S16) are strongest on the late picosecond–microsecond timescale and suggest an increase in conformational flexibility for this secondary structure element. See also Figures S9–16.

In TR-SFX, photoinduced differences in structure factor amplitudes, caused by population changes prepared by the pump laser pulse, are usually small. A further analysis is therefore implemented to extract these minor light-induced population changes from the structure factors obtained for each time point, once key regions of interest are identified from the DED maps above (e.g., the chromophore region). This method is based on the background subtraction estimation implemented by Pearce and colleagues^53^ and consists of generating maps, called here *W*Δ*F*_max_ maps, from the measured illuminated and dark structure factors and reference phases. These maps differ from the DED maps above in that a background subtraction factor (0 < *N*_bg_ < 1) is estimated to generate a *w*(|*F*_illuminated_| – *N*_bg_ × |*F*_dark_|) weighted map (Figures S9–S12 and Supporting Information Procedures Section 1). The *W*Δ*F*_max_ map for the 100 ps time point is shown in Figure 5a and illustrates the efficacy of the background estimation: the *cis anti* photoproduct is clearly present, with electron density at +1.8σ outlining both rings and a large portion of the isomerized double bond. WΔF_max_ maps for 300 fs, 600 fs, 900 fs, and 1 μs also confirm the formation of the *cis anti* species (Figure S11). The 600 and 900 fs *W*Δ*F*_max_ maps support the assignment of Peak 1 to a *trans* intermediate. The electron densities of these two maps are very similar and present four key characteristics: (i) the presence of peak 1, (ii) an elongated and uncentered peak where the *cis anti* chlorine is positioned, which is in contrast with the round, centered features visible in the WΔF_max_ maps from the later time points, (iii) electron density that fills the *cis anti* chromophore phenol ring, again in contrast with the 100 ps and 1 μs maps that show instead a clear outline of the chromophore phenol ring where its center is empty, and (iv) features that suggest a tilt of the imidazolinone ring oxygen toward the phenol ring (Figures S11 and 12). Though assignment is complicated by the likely presence on both *trans-FS* and *cis anti* species, refinement of coordinates to 600 and 900 fs *W*Δ*F*_max_ maps leads to two chromophore configurations that are almost superimposable (Figure S13). We therefore suggest that an intermediate state, *trans-FS*, accumulates with a 600–900 fs timescale. Because the 900 fs data appears to have less contamination from the *cis anti* species than the 600 fs data, we take the coordinates refined from the 900 fs *W*Δ*F*_max_ map as the proposed *trans-FS* structure. This chromophore configuration is still *trans* but more twisted (the torsion angles for the refined structure are ϕ ≈ 33° and τ ≈178°); such change is accompanied by a movement of the imidazolinone ring by about 0.5 Å, which is particularly noticeable from the shift in the electron density of its oxygen (Figure 4). Comparable ultrafast formation of a twisted *trans* chromophore in protein photoisomerization has been observed before in rsEGFP2^45^ and PYP^54^ and has been assigned to the excited state. Similarly, we also suggest assignment of *trans-FS* to an excited-state species on the basis of QM-MM calculations (see below). *W*Δ*F*_max_ maps thus allow us to model photoinduced species more effectively than the DED maps in Figure 4. Through them, we confirm positioning of the *cis anti* configuration as well as the structure of the *trans-FS* intermediate (Figures 5 and S10–S13). After establishing coordinates through *W*Δ*F*_max_ maps, we use the raw data once again to refine occupancies of new chromophore conformations for each time point (Figure S14). We note that clear identification of photoproduct and intermediate species here is greatly helped by the higher X-ray scattering cross-section of chlorine, both in DED and in *W*Δ*F*_max_ maps, highlighting a further advantage of introducing a heavy atom substituent in the chromophore structure. The confident assignment of *cis anti* formation at ultrafast time points provides strong evidence for the HT pathway in this rsEGFP2 construct. The observation of photoisomerization at femtosecond delays is likely associated with the relatively high primary quantum yield of this protein. The significant transient concentration and large displacement of the chromophore in the reactive pathway of Cl-rsEGFP2 strongly contribute to the light-induced differences, while additional contributions, such as vibrational coherences, will be below this level.

#### Protein Residue/Structure Changes

Strong DED features in our data are almost entirely concentrated on the chromophore (Figure 5b). To investigate the presence of any other motions across the protein structure that are associated with chromophore twisting and photoisomerization, we use the analysis and scripts from Wickstrand et al.^55^ and plot DED signals along the protein sequence for every time point collected (Figure 5c). Data from 600 fs, 900 fs, 100 ps, and 1 μs delays display the best crystallographic statistics (Table S1), so we concentrate on these to identify the areas of further protein structural change. Aside from the chromophore (the OHD ligand in the structures) that, as expected, presents the largest light-induced differences, there are two regions of notice: the central α-helix (residues 58–67) and β-strand 7 (residues 146–152), which interfaces with the chromophore phenol ring. Figures S15 and 16 show the DED on specific residues of these two secondary structure elements for different time points. Signals on the central α-helix suggest an overall downward motion of the region by around 1.4 Å if the distance between the strong positive/negative features on Leu65 and Thr63 is measured. A downward movement of this helix was previously observed in 1 ps DED maps of non-chlorinated rsEGFP2^45^ and was attributed to a tilt of the imidazolinone ring upon chromophore twisting. Similarly, here the prevalence of these signals in the sub-picosecond time points indicates that this motion is closely associated with the ultrafast photoisomerization of the bound chromophore or formation of the twisted *trans-FS* intermediate.

Negative DED signals on the side chains and backbone of β-strand 7 (β7) residues Asn147, Asn150, and Val151 are superimposable between the 100 ps and 1 μs datasets and hint at increased structural flexibility of this secondary structure element at later time points. Conformational fluctuations involving mainly β7, as well as that side of the β-barrel (β7–10), have been reported on the nanosecond–millisecond timescale for multiple GFP-like proteins,^56−61^ including rsEGFP2.^61^ This structural relaxation and plasticity that arises after photoactivation has been hypothesized to be important for efficient photoswitching between distinct protein conformations based on NMR, spectroscopy, and MD results.^57,59,60^ We have now obtained time-resolved crystallographic evidence that supports the development of β7 dynamics starting from 100 ps and peaking in the microsecond regime (Figures 5 and S16) in Cl-rsEGFP2. In our maps, β7 motions are accompanied by a flip of His149 from its characteristic OFF conformation to its ON conformation^44,45,48^ (Figure S16). The negative density on the side chain of His149 that indicates the flip to its ON conformation becomes clear at 1 μs, agreeing with recent time-resolved multiple-probe infrared spectroscopy (TRMPS-IR) data^61^ that assign His149 movement to a 42 ns time constant following ultrafast chromophore photoisomerization.

### Ultrafast Visible Transient Absorption Spectroscopy of Cl-rsEGFP2 Solutions

**Figure 6:**
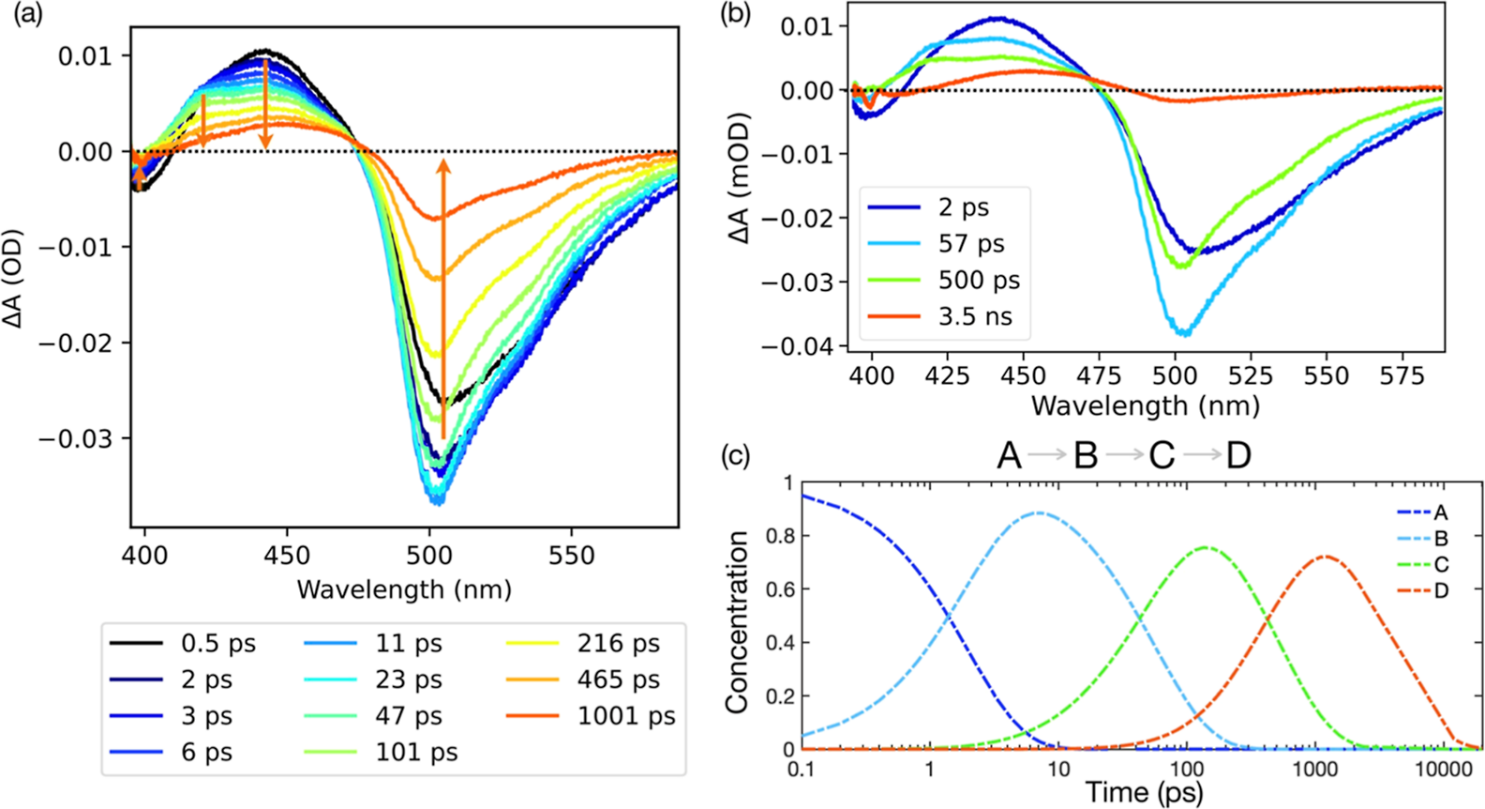
Femtosecond Cl-rsEGFP2 TA data. (a) Transient difference absorption spectra recorded at different pump–probe time delays after a femtosecond laser excitation (400 nm) starting from the Cl-rsEGFP2 OFF state. (b) Components fitted through global analysis of the data shown in (a). (c) Raw concentration profiles for the four components globally fitted using a sequential model. See also Figures S17 and S18.

We monitored the spectral changes induced by 400 nm laser excitation in solutions of Cl-rsEGFP2 in the OFF state (Figure 6a). The first photoinduced spectral changes we observe include an increase in absorption at 450 nm that peaks at around 500 fs (attributed to excited-state absorption—ESA), accompanied by ground-state bleach (GSB) in the 400 nm region and a broad stimulated emission (SE) signal around 530–540 nm. Global analysis groups these features in a first component that has a decay rate constant of 2 ps. Assuming a sequential model (see Experimental Methods Section), this first component decays into a second and then a third component that both present a new positive peak at 420 nm and respective decay rate constants of 57 and 500 ps. In the final 3.5 ns component, the absorption at 420 nm is absent, and the main positive peak present is once again at 450–460 nm (Figure 6b,c). While the wavelengths for GSB, 450 nm ESA, and SE present in Cl-rsEGFP2 time-resolved spectra closely match those of rsEGFP2 (Figures S17 and 18 and refs (44) and (61)), the transient picosecond positive peak at 420 nm is unique to the chlorinated construct (Figure S18). We also note that Cl-rsEGFP2 presents an interesting property of the stimulated emission band, which actually increases on the 2 ps timescale and then begins its decay with a 57 ps time constant. Such behavior is not observed in rsEGFP2 (Figures S17 and 18 and refs (44) and (61)). We discuss this and the 420 nm positive feature in the following sections.

### Quantum Chemical Analysis of HT Photoisomerization

**Figure 7:**
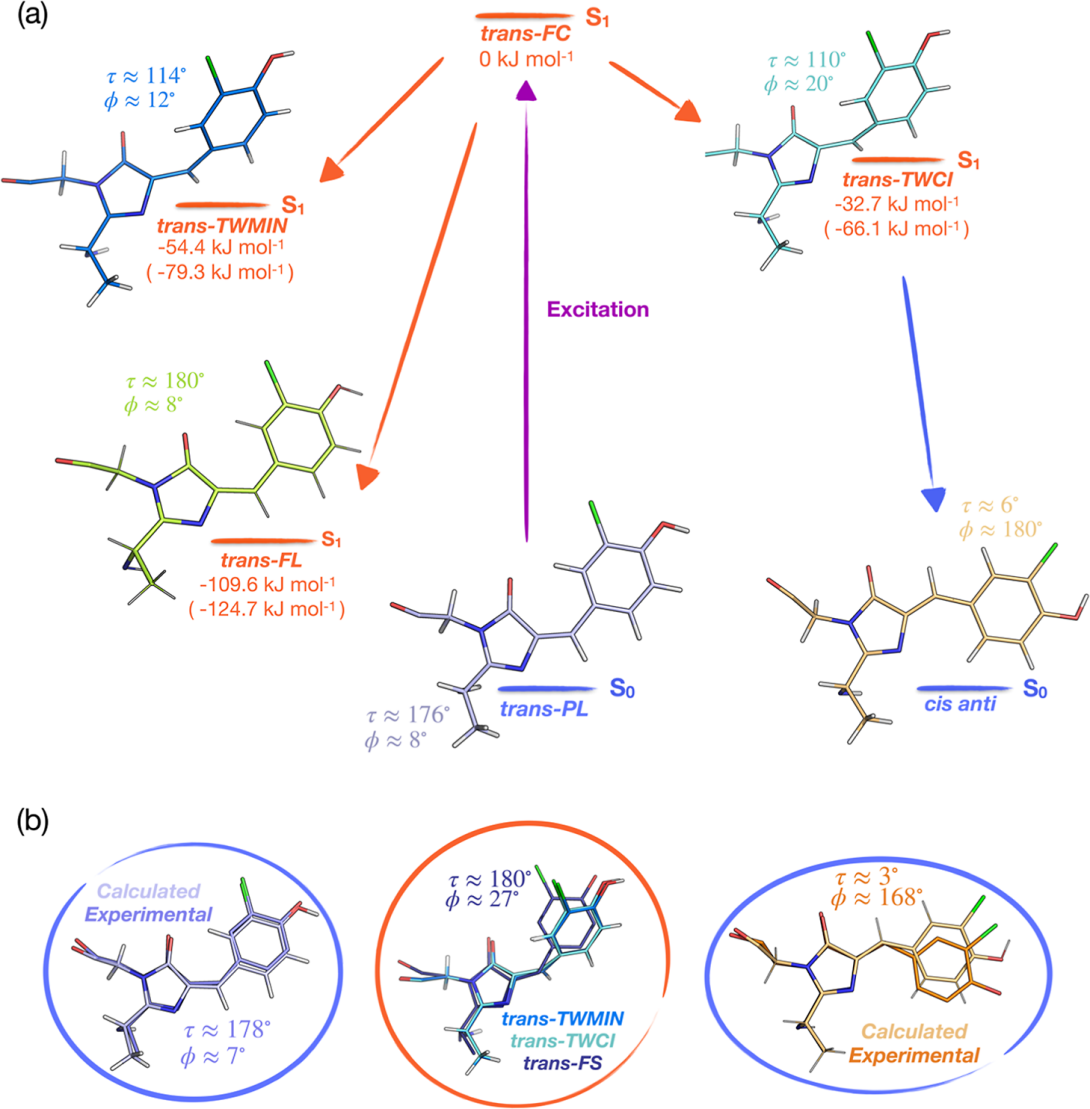
Comparison between experimental and calculated chromophore structures. (a) Molecular structures obtained through QM-MM modeling before and after CI crossing are shown with their energy arrangement relative to the Franck–Condon point and their respective torsion angles. Relative energy values computed at a lower (SA2-CASSCF(12,11)/3-21G//Amber03) and higher (XMCQDPT2/SA6-CASSCF(12,11)/cc-pVDZ//Amber03—in parenthesis) level of theory are stated (see also Section 4 in the Supporting Information Procedures). The photoexcited *trans-PL* species isomerizes in the protein binding pocket to the planar *cis anti* via HT, passing through a highly twisted conformation near the CI (*trans-TWCI*). An excited-state twisted minimum structure (*trans-TWMIN*) and a fluorescent *trans* minimum (*trans-FL*) are also found. (b) The calculated conformations are compared to the planar *trans anti* and *cis anti* species, as well as to the twisted *trans-FS* intermediate, refined from TR-SFX data. See also Figure S19.

To provide additional support for a HT photoisomerization pathway, we performed QM/MM optimizations at the SA2-CASSCF(12,11)/3-21G//Amber03 level of theory, in which we searched for conical intersections (CIs) associated with the OBF and HT isomerization mechanisms.^62^ At first, we optimized a planar *trans* S_1_ fluorescent minimum structure (*trans-FL*, Figure 7), which is further discussed below. Whereas no CI could be located along the OBF pathway, we could identify a minimum energy conical intersection (MECI) for a HT isomerization. Additionally, we performed re-computation of the energies in the optimized structures at the correlated XMCQDPT2/SA6-CASSCF(12,11)/cc-pVDZ//Amber03 level of theory. For a detailed description of these calculations, see the Supporting Information Procedure Section 4. It should be noted that the MECI is not a stationary point on the molecular potential energy surface but rather a geometry through which radiationless deactivation from the electronic excited to ground state occurs with high probability. The geometry at this CI features a ϕ torsion angle of 20° and a τ torsion angle of 110° (Figure 7b, *trans-TWCI*). Geometry optimizations in the electronic ground state confirm that this CI connects the neutral *trans* and *cis* chromophore conformers (Supporting Information Procedures Section 4). The value of τ in this *trans-TWCI* species is close to orthogonality and could suggest a stepwise (rather than concerted) nature to the HT pathway, though this additional detail is not resolved in our crystallographic data. At the levels of theory employed here, the *trans-TWCI* geometr*y* is lower in energy than the Franck–Condon point (Figure 7a). Thus, the results of our computations suggest that the HT dominates the isomerization process, while the OBF mechanism is suppressed.

In addition to the HT MECI geometry (*trans-TWCI*), we also identified a twisted S_1_ minimum geometry, with a ϕ torsion angle of 12° and a τ torsion angle of 114° (Figure 7b, *trans-TWMIN*), in line with the results from previous computations on the non-chlorinated HBI chromophore.^63,64^ This resembles the *trans-FS* structure refined from the 600–900 fs datasets. Because the energy required for photon absorption from this minimum into the S_5_ state is 434 nm and hence close to the transient absorption (TA) peak at 420 nm in the time-resolved TA spectroscopy measurements (Figure 6), the twisted S_1_ minimum could tentatively be assigned to the transient intermediate responsible for this absorption. A more confident assignment, however, would require TA measurements on crystalline samples to determine if the kinetics of the 420 nm absorption confirm accumulation of the *trans-FS* species in 600–900 fs.

## Conclusions

This study exemplifies the capability of the temporal and structural resolution available with serial crystallography at XFEL facilities. Through the combination of the SFX technique and the breaking of the rsEGFP2 chromophore symmetry via a heavy atom substituent, we resolve the primary photoproduct of the *trans*-to-*cis* photoisomerization and confidently assign it to be the outcome of a HT pathway. To our knowledge, this is the first experimental observation of HT photoisomerization in a protein on the femtosecond-to-picosecond timescale.

We additionally identify a twisted intermediate state at 600–900 fs that can potentially be assigned to an excited-state minimum on the basis of calculations. While we observe a possible correspondence between the calculated absorption spectrum of this intermediate and the 420 nm feature in our time-resolved TA spectra, we note that the sub-picosecond DED signal is dominated by the strongest displacement and highest ordering, and this may contribute only to the first decay component of the ESA and SE heterogeneous decay kinetics. The structural data obtained from TR-SFX reports on the thermodynamic distribution, displacements, cross-sections, and concentrations of ground and excited states in the protein at a specific point in time, while TA data is a kinetic description of the often heterogeneous electronic dynamics that depend on the spectroscopic selection rules. Because of this, the signal strengths and kinetics of TR-SFX and TA transients are not necessarily expected to be the same. In this case, we believe that further investigation, such as a pump–dump scheme, would be required for a confident connection between the crystallographic and spectroscopic signals we report. The current experimental evidence does not unequivocally support the involvement of the twisted *trans-FS* minimum in the reaction mechanism. In fact, given the observed sub-picosecond formation of the *cis anti* photoproduct, the presence of a twisted structure with a picosecond lifetime, and the predicted planar *trans*-S_1_ fluorescent minimum from calculation (*trans-FL* in Figure 7), we speculate that there are (at least) three major decay channels in Cl-rsEGFP2: (i) an ultrafast, energetically down-hill access to the MECI to form the HT photoproduct, (ii) relaxation into the *trans-FL* minimum, and (iii) twisted *trans* S_1_ minimum formation (*trans-FS*/*trans-TWMIN*). The last two cases can both promote fluorescence by transiently trapping the excited-state population and could provide an explanation for the long-lived fluorescent state observed in solution TA spectroscopy data (Figure 6).

The ability to track atomic displacements immediately following photon absorption is shown here to be essential for resolving the reaction pathway without having to rely on assumptions on the timescales of thermal bond rotations that can occur after photoactivation. The calculations presented and the use of room-temperature SFX suggest, in fact, that the HT is the only accessible photoisomerization coordinate in the Cl-rsEGFP2 chromophore pocket and that previously proposed crystal-packing constraints are most likely artifacts resulting from the cryo-trapping procedures used (Note S1 and ref (48)). Our results thus support the reasoning that the protein cage in Cl-rsEGFP2 hinders the OBF mechanism in favor of the HT.

What is the significance of this pathway? The presence of *cis anti* photoproduct signals as early as 300 fs introduces the possibility of this photoisomerization reaction being a vibrationally coherent process, as observed for rhodopsins.^65−69^ Vibrational dephasing times in GFP proteins have typically been reported to be within 1–2 ps,^70−72^ which does not exclude the 300 fs *cis anti* species to be the product of a vibrationally coherent photoisomerization. Further work, such as coherently controlling the photoisomerization and tracking its yield,^67^ would be required to investigate this. Interestingly, Adam and colleagues report that rsEGFP2 mutants in which the available binding pocket volume is decreased switch to their ON state much more efficiently than mutants where the available binding pocket volume is increased.^52^ It will be valuable to investigate whether a HT reaction pathway dictated by a volume-constraining protein cage is responsible for this increased switching capability and whether coherent CI crossing is important for the efficiency of photoactivation. Understanding the details of the photoactivation process such as these holds significant promise for the rational engineering of fluorescent proteins like Cl-rsEGFP2.^14,15,73−75^ Serial femtosecond crystallography has also allowed us here to trace the effects of the early photoisomerization event to secondary structure rearrangements that occur later in time. This data provides time-stamped structural insight that integrates and reconciles previous observations from other experimental and computational techniques; it furthers our understanding of the dynamics that enable protein function through photoactivation and develops our ability to intelligently manipulate them for improved performance.

## Experimental Methods

### 2021 SFX Sample Preparation, Data Collection, and Setup

#### Protein Expression, Purification, and Crystallization

Recombinant expression was performed in BL21 (DE3) cells with a pET-15b vector for the rsEGFP2 gene and a pUltra vector containing the 3-chlorotyrosine amber suppression codon. The latter vector was used instead of the pEVOL vector described by Chang et al.^48^ Large-scale bioreactor fermentation proceeded with a 50 L culture in Terrific Broth Modified (46.7 g/L) containing 100 mg/L ampicillin, 50 mg/L spectinomycin, 10.1 g/L glycerol, and 0.1 g/L 3-chlorotyrosine at 37 °C. After reaching an OD_600_ nm of ≈2, the culture was cooled to 20 °C, and induction was carried out by the addition of 0.24 g/L isopropyl β-D-1-thiogalactopyranoside. The culture was grown overnight for 17 h, harvested by pelleting, and stored at −20 °C.

Thawed cell pellets were washed with 50 mM Tris–HCl pH 8.0, 100 mM NaCl, 0.1 mg/mL DNase I, and 1 tablet of EDTA-free protease inhibitors per 100 mL of cells. Lysis was carried out at 4 °C with two passes at 35 kpsi in a T5 Cell disruptor (Constant Systems, UK). The lysate was centrifuged at 142 000 × *g* and 4 °C for 30 min before incubating the cell free extract with Ni-NTA resin for 45 min while shaking. After loading the slurry onto a glass column, the bound protein was washed with 2 column volumes of the above buffer containing 20 mM imidazole, followed by 2 column volumes containing 30 mM imidazole. The protein was then eluted with 200 mM imidazole, concentrated, and buffer-exchanged into 50 mM Hepes pH 7.5 and 100 mM NaCl using Vivaspin 10 kDa MWCO filters at 4 °C. The final purification step was carried out with a Superdex S75 gel filtration resin, and the main fractions were concentrated and finally buffer-exchanged into 50 mM Hepes pH 7.5 and 200 mM NaCl. The protein was either stored at −20 °C or used for crystallization.

Seeded batch crystallization proceeded with a final protein concentration of 20–24 mg/mL in a buffer consisting of 75 mM Hepes pH 8, 20 mM NaCl, and 1.1–1.3 M ammonium sulfate at 20 °C. Cuboid crystals with typical dimensions of 4–8 × 4–8 × 20–80 μm were obtained after 3 days.

#### Sample Preparation and Injection

Before injection, the crystals were buffer-exchanged into 25% PEG buffer and size-selected using a 50 μm filter and subsequently a 30 μm filter. Crystals were then delivered to the interaction region using a SACLA’s droplet-on-demand injector (MICROJET)^76^ and an 80 μm nozzle. Volumes of around 250 μl of crystal slurry were reverse-loaded through the jetting aperture to reduce the risk of blockages. The crystal slurry density was between 2 and 5 × 10^7^ crystal/mL. The driving voltage for the injector piezo was normally 110 V, and current pulses were 100 μs long. Throughout the experiment, a hit rate between 30 and 60% was maintained.

#### SFX Data Collection and Optical Setup

2021 data at SACLA^77^ was collected at Experimental Hutch 2 of BL3. The detector used was the MPCCD-phase III detector,^78^ and the detector distance was refined to 49.2 mm using unit cell distributions. The XFEL was operated with a repetition rate of 30 Hz, a photon energy of 10.5 keV, and a focal spot of ≈1.5 μm in full width at half-maximum (FWHM).

Crystals were pre-illuminated by direct illumination of the glass tip of a droplet injector with an unfocused (≈2 mm beam size) 100 mW 488 nm CW laser. Offline testing with the same laser and crystal concentration demonstrated maximum conversion after 2 s of illumination well within the ≈8 s transit time of the illuminated part of the jet during normal operation. 400 nm optical pump pulses were created by second harmonic generation of the SACLA synchronized femtosecond laser system using a 100 μm BBO crystal. The pulse length of 800 nm fundamental was measured to be 75 fs by autocorrelation. 7.5 μJ pump pulses were focused onto the interaction region using a 300 mm lens to give a spot size of 130 μm (FWHM) and a corresponding energy density of 0.5 mJ/mm^2^. Light and dark data were interleaved in a 5:1 ratio. A spatial overlap between the optical and XFEL beams was confirmed using a 50 μm Ce:YAG crystal. The same crystal was used to perform a cross-correlation and find a temporal overlap; the cross-correlation was fitted using the same methods described in (79). A temporal jitter between the optical and X-ray pulses was monitored using the SACLA timing tool system;^80^ however, recent upgrades to the synchronization of the optical laser and XFEL^81^ showed that the measured jitter measured was ≈50fs (FWHM) less than the instrument response of the measurement (≈100 fs), indicating that post-processing sorting of the TR-SFX data was unnecessary. The new system also actively corrects for slow timing drift between optical and XFEL beams, which was confirmed by cross-correlation measurements at the start and end of data collection (see SI for more details). Following data analysis methods are detailed in Section 1 of the Supporting Information Procedures.

### Femtosecond TA Spectroscopy

Femtosecond TA data was measured using the system described in Lincoln et al^82^ White light probe pulses were generated using filamentation in a CaF_2_ glass window. 400 nm pump pulses were generated by doubling the fundamental 800 nm (Hurricane, Spectra Physics) in a 100 μm-thick SHG-BBO (Eksma optics). The pump pulses were focused onto the sample using an AR-coated f = 500 nm lens (Thorlabs). The pump energy density was ≈0.02 mJ/mm^2^. A 20 μl solution of Cl-rsEGFP2 was mounted in a liquid flow cell (Harrick Scientific Products Inc) between a 1 mm (front) and a 2 mm (back) sapphire windows (Crystran Ltd) using a 25.6 μm spacer. The sample concentration was chosen to give an OD of ≈0.1 at the 400 nm peak of the OFF state (Figure 2c). The sample was pre-illuminated using an unfocused 50 mW 488 nm diode laser to ensure presence of the target OFF state and was continuously translated during data collection. Using the Ultrafast Spectroscopy Modeling Toolbox,^83^ a second-order polynomial was fitted to the pump coherent artefact and was sufficient to correct the majority of the inherent spectral chirp of the white light probe. A sequential model was then applied in the Toolbox for SVD analysis and global fitting. Application of a parallel model leads to overfitting, which is made clear by the presence of compensating amplitudes in the fitted components (not shown).

## Supporting Information Available

Frame data processing and crystallographic analysis; crystallographic map generation procedures; 2019 rsEGFP2 OFF state and cryo-trapping experiment; quantum chemical modeling procedures; anomalous crystallographic maps for confirmation of chlorine atom; and quantum yield calculations for Cl-rsEGFP2 (PDF)
