# Supplementary material for: Serial Femtosecond Crystallography Reveals that Photoactivation in a Fluorescent Protein Proceeds via the Hula Twist Mechanism
Source: J Am Chem Soc. 2023 Jul 7;145(29):15796–808. doi: 10.1021/jacs.3c02313 (PMC10375524; doi:10.1021/jacs.3c02313)
Supplement: Supplementary file 1 — ja3c02313_si_001.pdf [file ja3c02313_si_001.pdf]

Serial Femtosecond Crystallography Reveals that Photoactivation in a Fluorescent Protein Proceeds via the Hula Twist Mechanism - Supporting Information

Authors:

Alisia Fadini[1], Christopher D.M. Hutchison[1], Dmitry Morozov[2], Jeffrey Chang[3], Karim Maghlaoui[1], Samuel Perrett[1], Fangjia Luo[4], Jeslyn C.X. Kho[1], Matthew G. Romei[5], R. Marc L. Morgan[1], Christian M. Orr[6], Violeta Cordon-Preciado[1], Takaaki Fujiwara[7], Nipawan Nuemket[4,8], Takehiko Tosha[4], Rie Tanaka[4,8], Shigeki Owada[4,9], Kensuke Tono[4,9], So Iwata[4,8], Steven G. Boxer[5], Gerrit Groenhof[2], Eriko Nango[4,7*], Jasper J. van Thor[1*]

Affiliations:

[1] Department of Life Sciences, Faculty of Natural Sciences, Imperial College London, London, SW7 2AZ, UK

[2] Nanoscience Center and Department of Chemistry, University of Jyväskylä, Jyväskylä, 40014, Finland

[3] Department of Physics, Stanford University, Stanford, CA 94305, USA

[4] RIKEN SPring-8 Center, 1-1-1 Kouto, Sayo, Sayo, Hyogo, 679-5148, Japan

[5] Department of Chemistry, Stanford University, Stanford, CA 94305, USA

[6] Diamond Light Source Ltd, Harwell Science and Innovation Campus, Didcot, OX11 0DE, UK

[7] Institute of Multidisciplinary Research for Advanced Materials, Tohoku University, 2-1-1 Katahira, Aoba, Sendai, Miyagi, 980-8577, Japan

[8] Department of Cell Biology, Graduate School of Medicine, Kyoto University, Yoshidakonoe, Sakyo, Kyoto, 606-8501, Japan

[9] Japan Synchrotron Radiation Research Institute, 1-1-1 Kouto, Sayo, Sayo, Hyogo, 679-5198, Japan

Supporting Figures and Tables

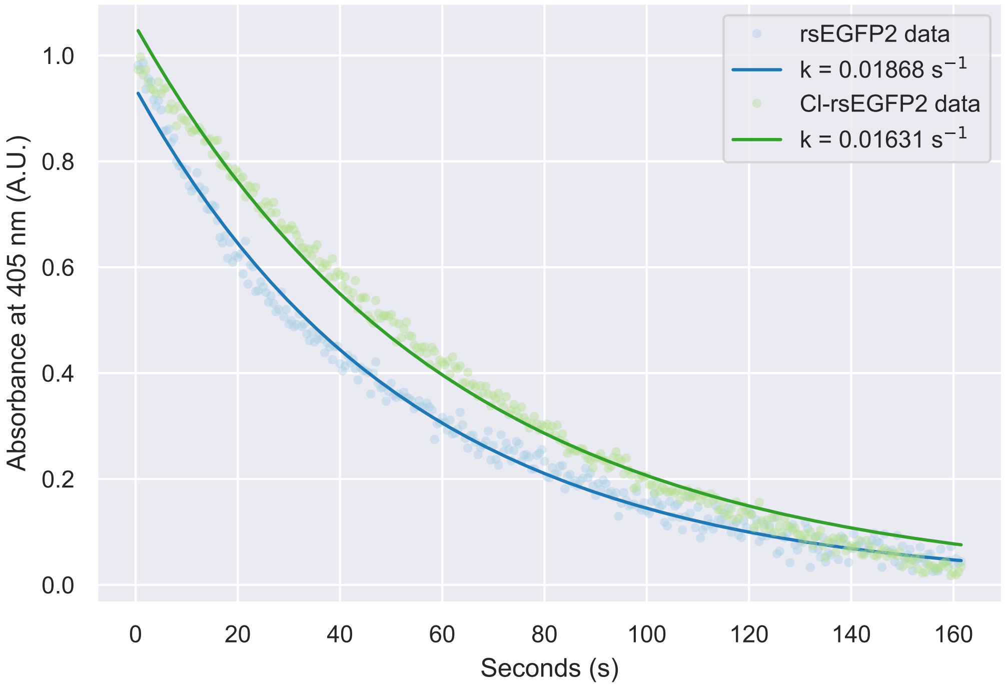

Figure S1. OFF-to-ON switching rates for chlorinated and unchlorinated rsEGFP2 constructs measured under the same conditions. An exponential decay of the form $y = Ae^{-kt}$ was fitted to obtain the rate k for each construct. Related to Figure 2.

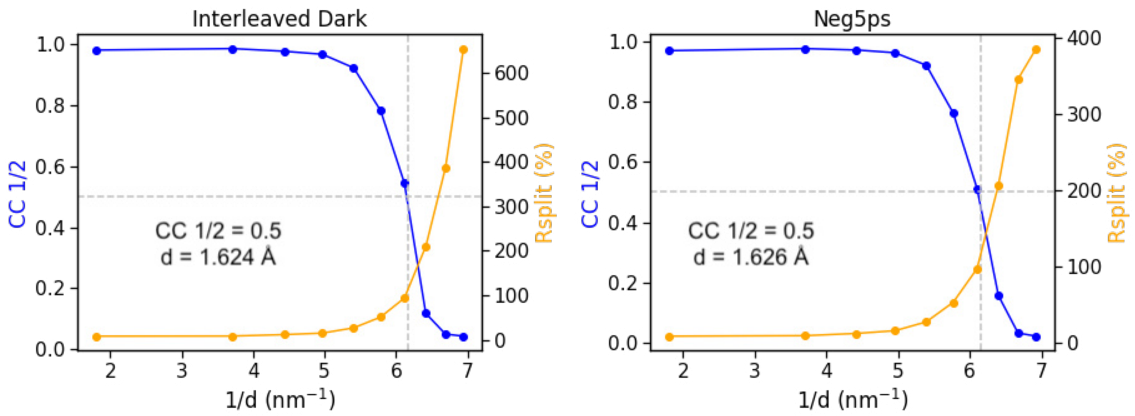

Figure S2. Choice of resolution cutoff from the dark SFX data. A $CC_{1/2}$ of 0.5 was chosen, corresponding to ≈ 1.63 Å for both the interleaved dark and -5ps datasets.

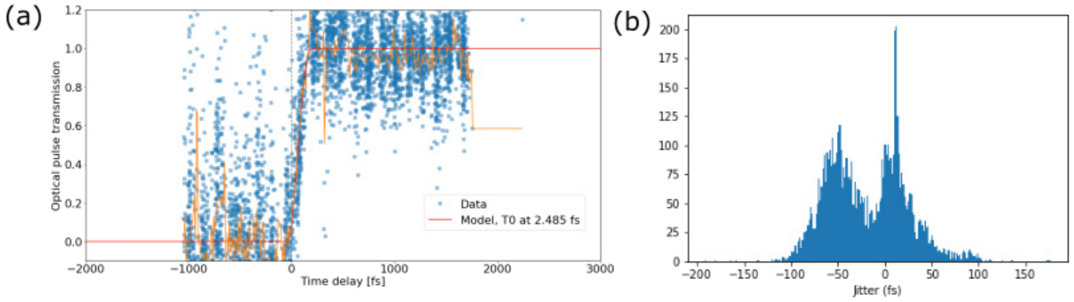

Figure S3. (a) Cross-correlation between the laser pump and XFEL probe for time zero determination and **(b)** jitter analysis for the SACLA 2021 experiment. Related to Figure 4.

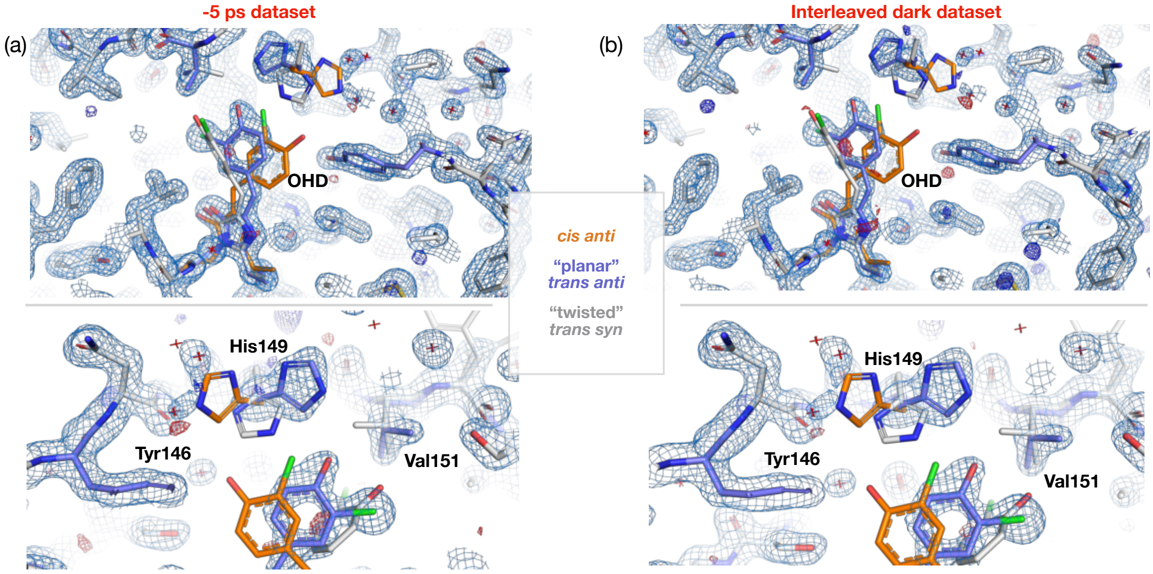

Figure S4. 2mFo-DFc maps, shown at +1.8σ, computed from the -5 ps **(a)** and interleaved dark **(b)** datasets and the refined dark structure (PDB 8A6G). mFo-DFc maps are shown in red and green at -3σ and +3σ respectively. The top and bottom panels show two different views of the chromophore (OHD) region. The three chromophore populations modeled in the dark state, planar *trans anti* (*trans-PL*), twisted *trans syn* (*trans-TW*), and *cis anti*, are shown in distinct colors with the corresponding interacting residue conformations. Related to Figure 3.

(a)

(b)

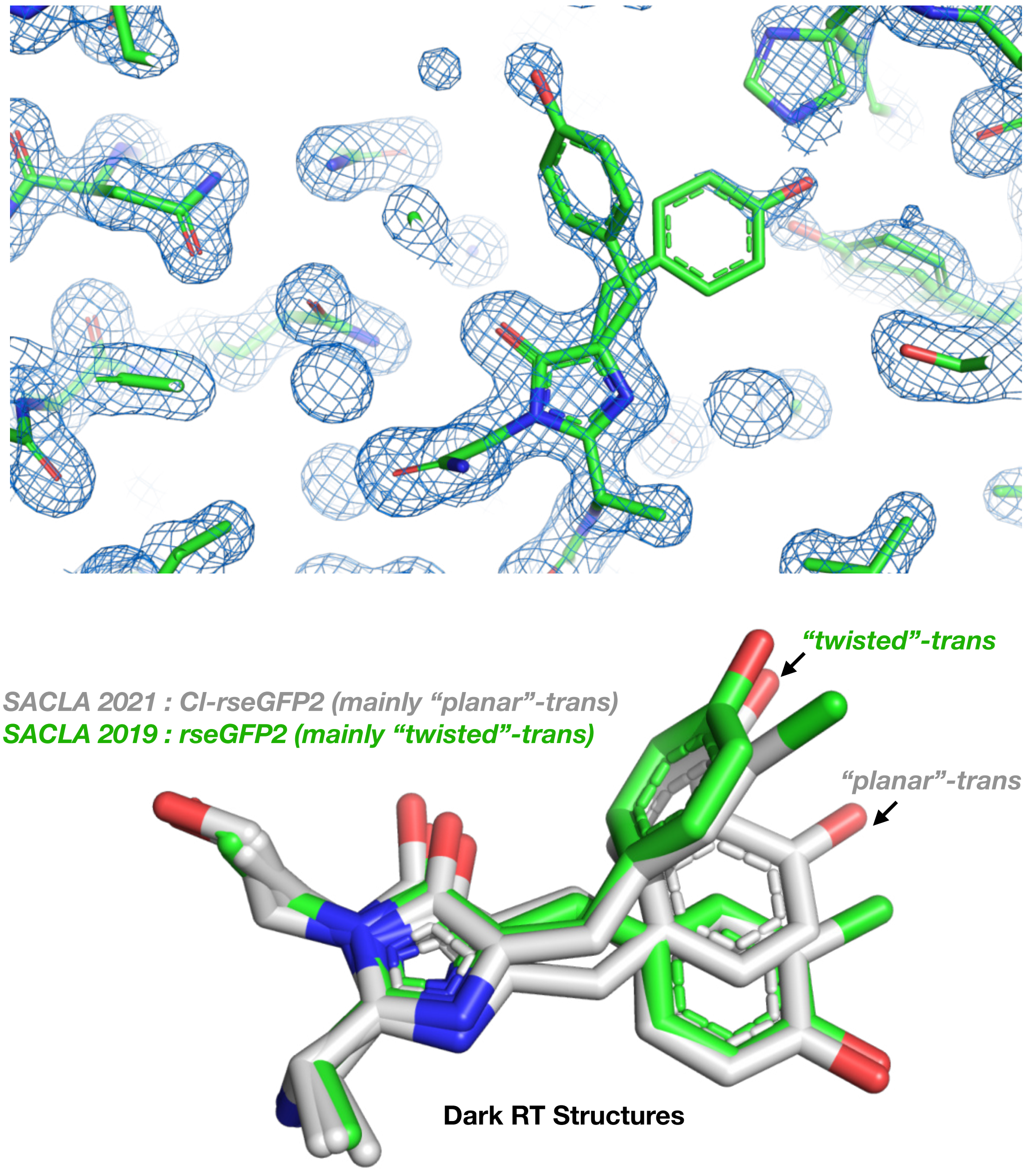

Figure S5. (a) 2mFo-DFc map, shown at 1.8σ, computed with the reflections from dark data collected on rsEGFP2 (unchlorinated) at SACLA in 2019 and the refined dark structure (PDB 8A7V). The prevalent chromophore conformation is the *trans*, though a small population of *cis* is also refined. **(b)** Superposition of the refined dark room temperature (RT) structures for the unchlorinated rsEGFP2 (SACLA 2019, green) and Cl-rsEGFP2 (SACLA 2021, gray). It is highlighted how the major chromophore *trans* configuration differs between the two: rsEGFP2 is found in the twisted *trans* configuration (*trans-TW*), while Cl-rsEGFP2 is mainly in the planar *trans* configuration (*trans-PL*). Both datasets present minor *cis* chromophore conformations. Related to Figure 3.

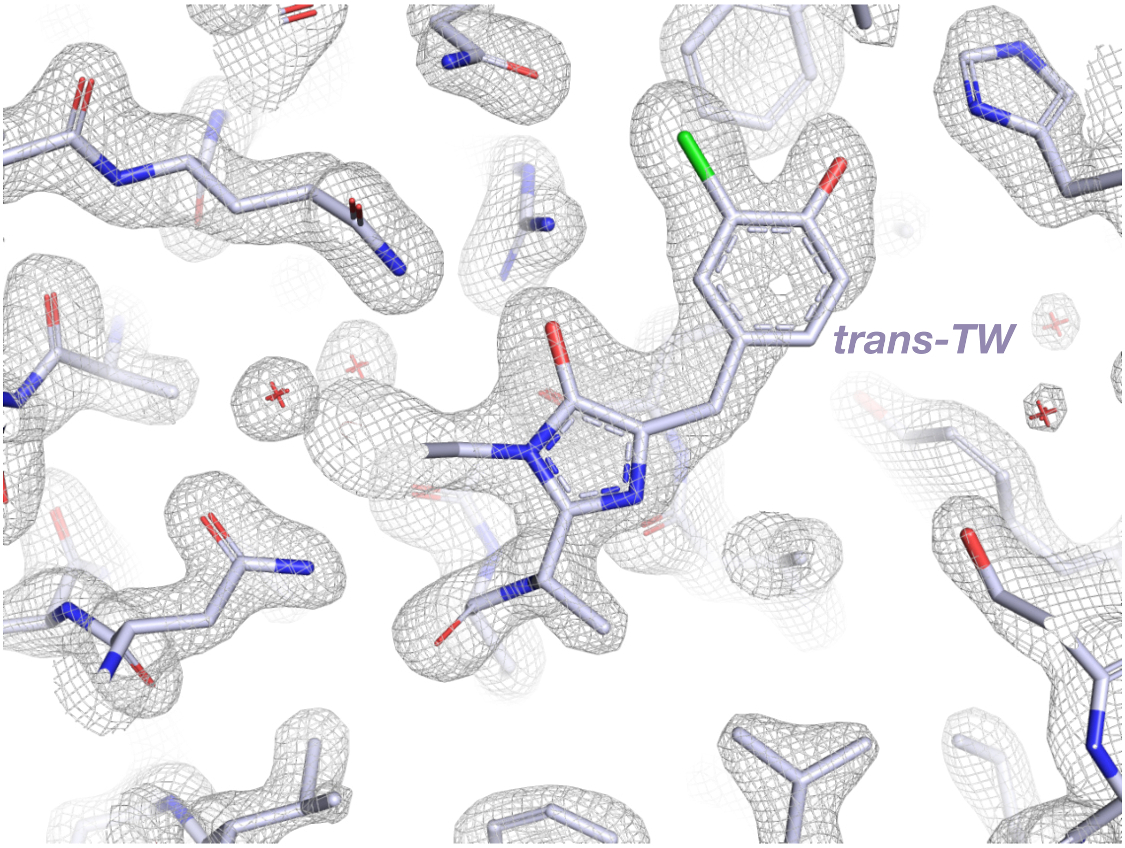

Figure S6. 2mFo-DFc map, shown at 1.8σ, computed with the reflections from data collected on Cl-rsEGFP2 at SSRL after illumination and dehydration and the refined structure (PDB 8A83). The prevalent chromophore conformation is *trans-PL*. Related to Figure 3.

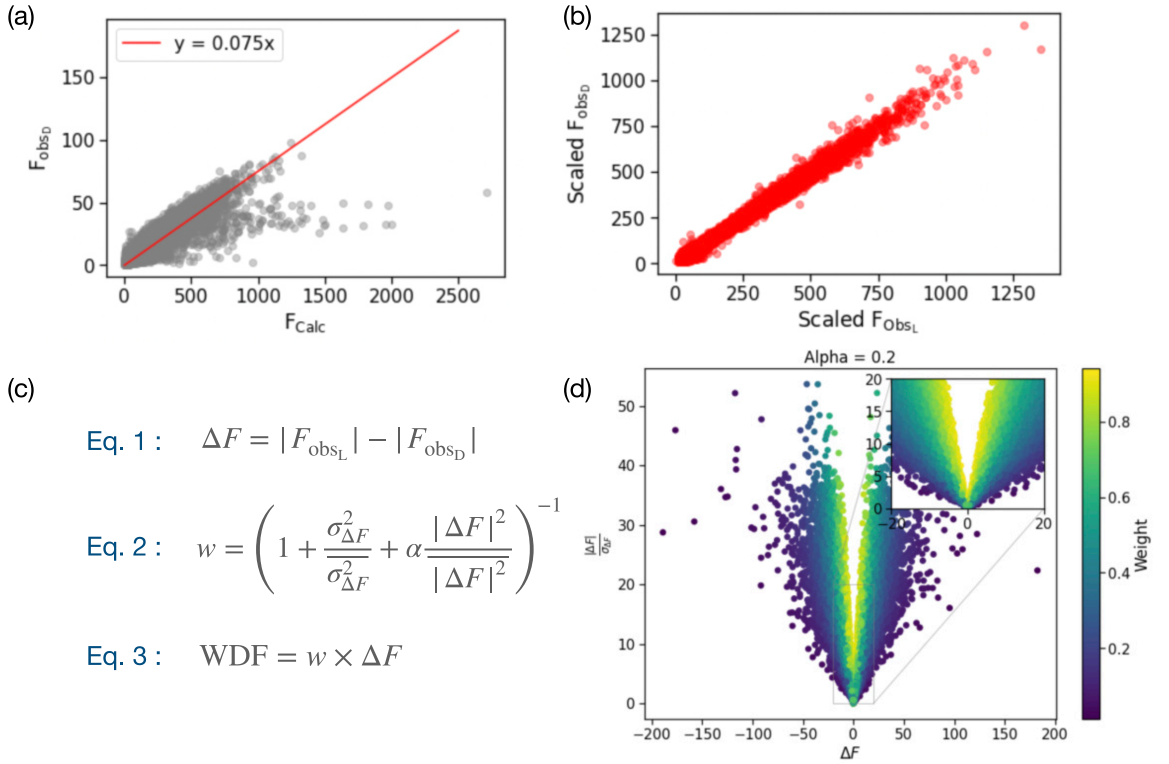

Figure S7. (a) Example of scale factor determination. The structure factors from the -5 ps dataset (F_{obs_D}) are plotted against structure factors from the refined dark model (F_{calc}) and a line of the form y = *m*x is fitted to the data. For the dark structure factors, *m* = 0.75. In the same manner, the light structure factors for every time point are scaled to F_{calc}. **(b)** Example of the result of scaling on dark (F_{obs_D}) and light (F_{obs_L}) structure factors (here the 100 ps dataset is shown). For the data presented in this work, this simple scaling procedure is sufficient to yield highly correlated light and dark structure factors. **(c)** Mathematical procedure to obtain weighted difference structure factors (WDF) from dark and light structure factors. $\sigma_{\Delta F}$ is the structure factor standard deviation and the term α reduces the contribution of any single ΔF. **(d)** Weights applied to the 100 ps dataset for α=0.2, shown as an example. Difference structure factors that are large or measured with a higher error are given progressively lower weight. Related to Figure 4.

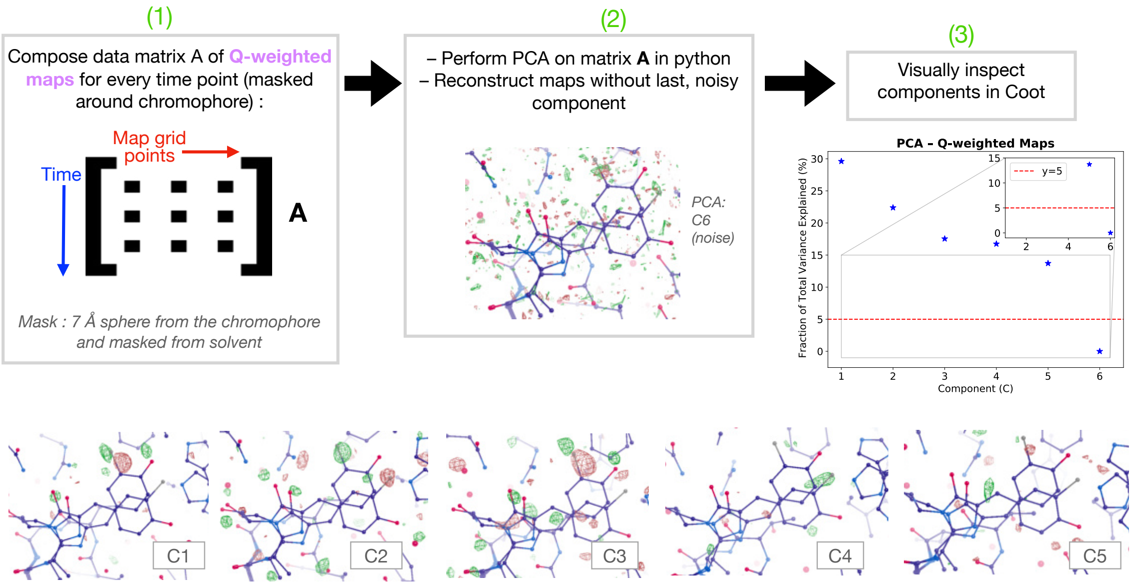

Figure S8. The pipeline implemented to denoise Q-weighted maps through PCA is shown. Each map from a collected time point is a row in the data matrix A (6 time points total: 300 fs, 600 fs, 900 fs, 5 ps, 100 ps, 1 μs) and PCA is performed using the sci-kit learn package in python. The last (C6) component found explains less than 5% of the total variance in the data and, upon visual inspection, appears as noise. The first five components are shown in the bottom panels at +/-3.5σ. Related to Figure 4.

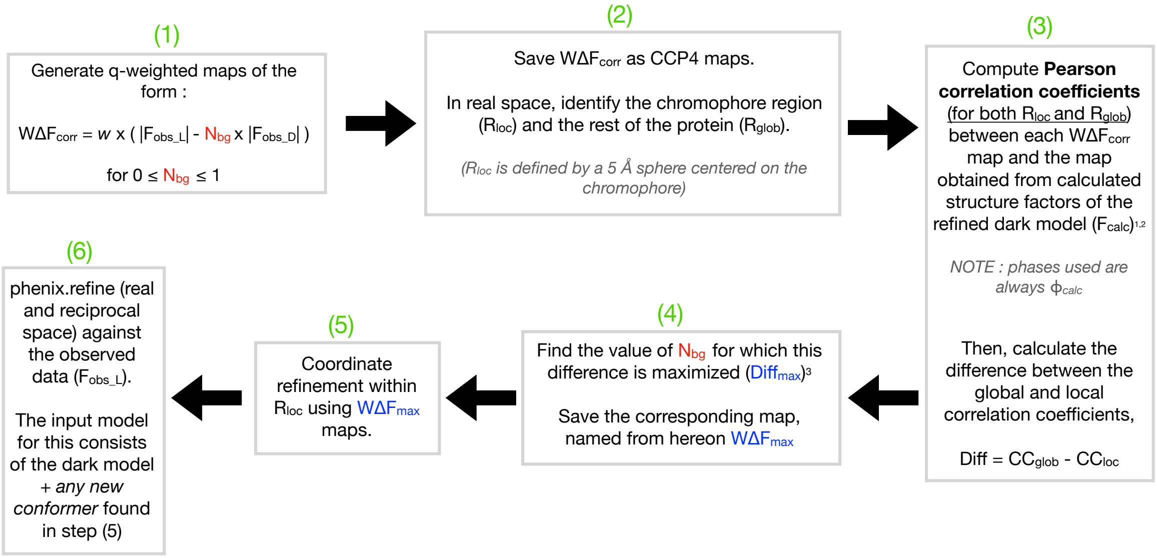

1) Terwilliger, T. C. & Berendzen, J. Bayesian difference refinement. *Acta Crystallogr. D* **52**, 1004–1011 (1996).
2)Pandey, S., Bean, R., Sato, T. *et al.* Time-resolved serial femtosecond crystallography at the European XFEL. *Nat Methods* **17,** 73–78 (2020).
3) Pearce, N. M. et al. A multi-crystal method for extracting obscured crystallographic states from conventionally uninterpretable electron density. Nat. Commun. 8, 15123

Figure S9. Schematic of the steps involved in the generation of background subtracted maps (WΔF$_{\mathrm{max}}$ maps). Related to Figure 5.

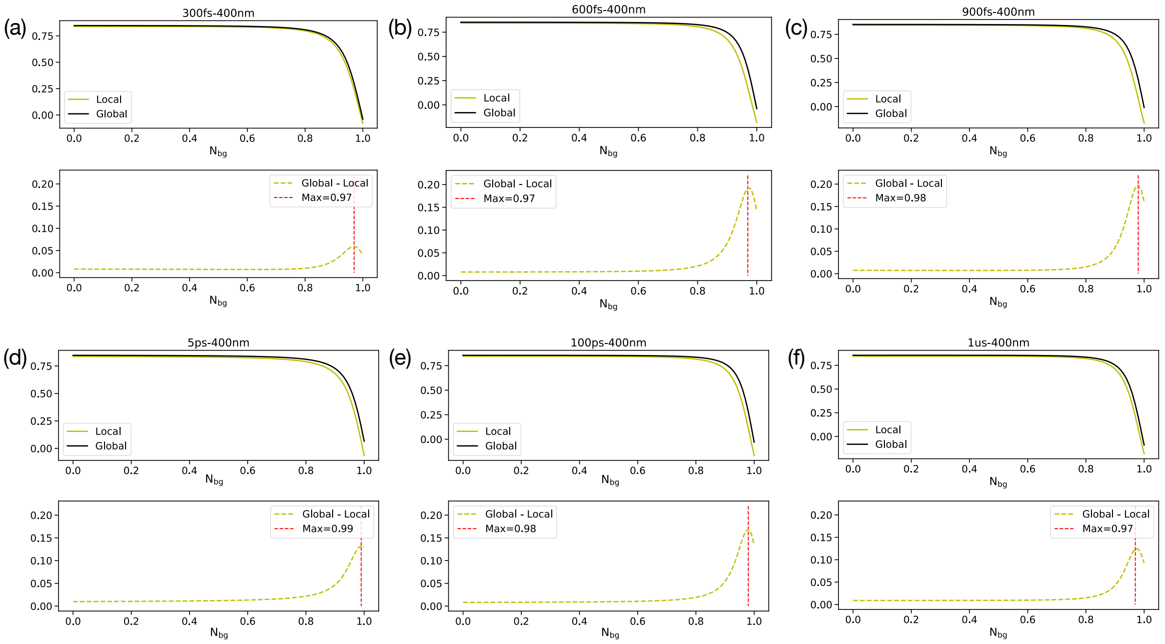

Figure S10. Identification of the appropriate background subtraction factor (N_{bg}) for the $W\Delta F_{max}$ map of each timepoint **(a-f)**. The top panels plot the correlation coefficient between the original and the background subtracted map for different N_{bg} values calculated across the entire protein structure (global) and a specific 5 Å sphere centered on the chromophore (local). The bottom panels plot the difference between the global and local lines and highlight the value of N_{bg} for which the difference in correlation coefficients is maximized. Related to Figures 4 and 5.

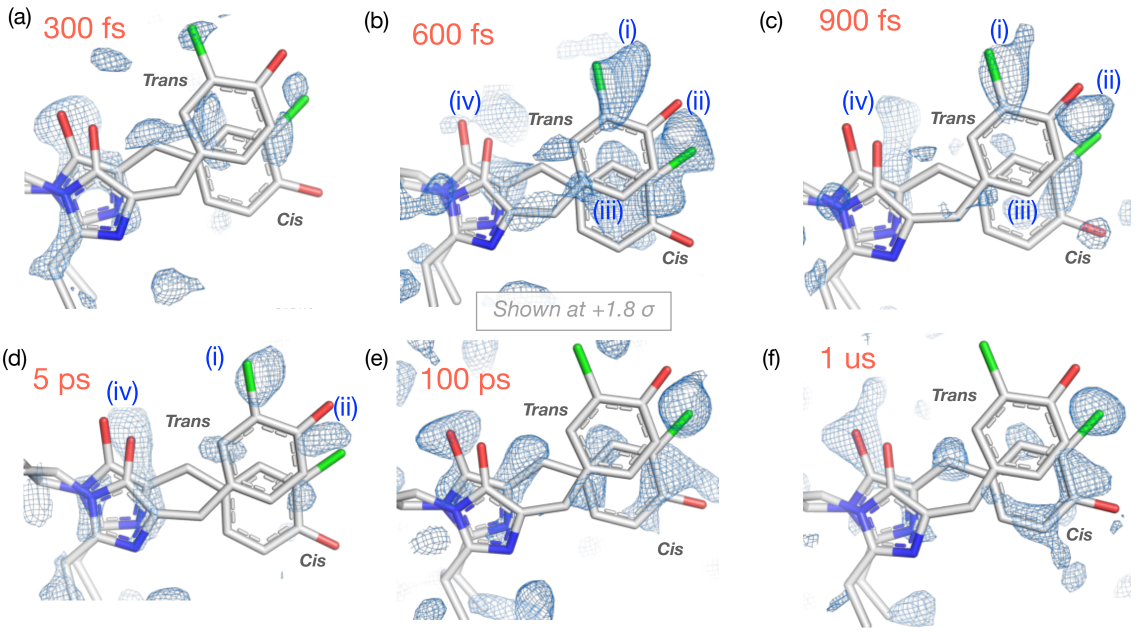

Figure S11. $W\Delta F_{max}$ maps generated using the method described in this work and illustrated in Figure S9. The following four characteristics, which we attribute to the presence of a femtosecond intermediate, are marked in the 600 fs and 900 fs maps: (i) the presence of Peak 1 (ii) an elongated and uncentered peak where the *cis anti* chlorine is positioned, which is in contrast with the round, centered features visible in the $W\Delta F_{max}$ maps from the later time points (iii) electron density that fills the *cis anti* chromophore phenol ring (iv) features that suggest a tilt of the imidazolidone ring oxygen towards the phenol ring. Though less pronounced, these features are also identifiable in the 5 ps map. Related to Figures 4 and 5.

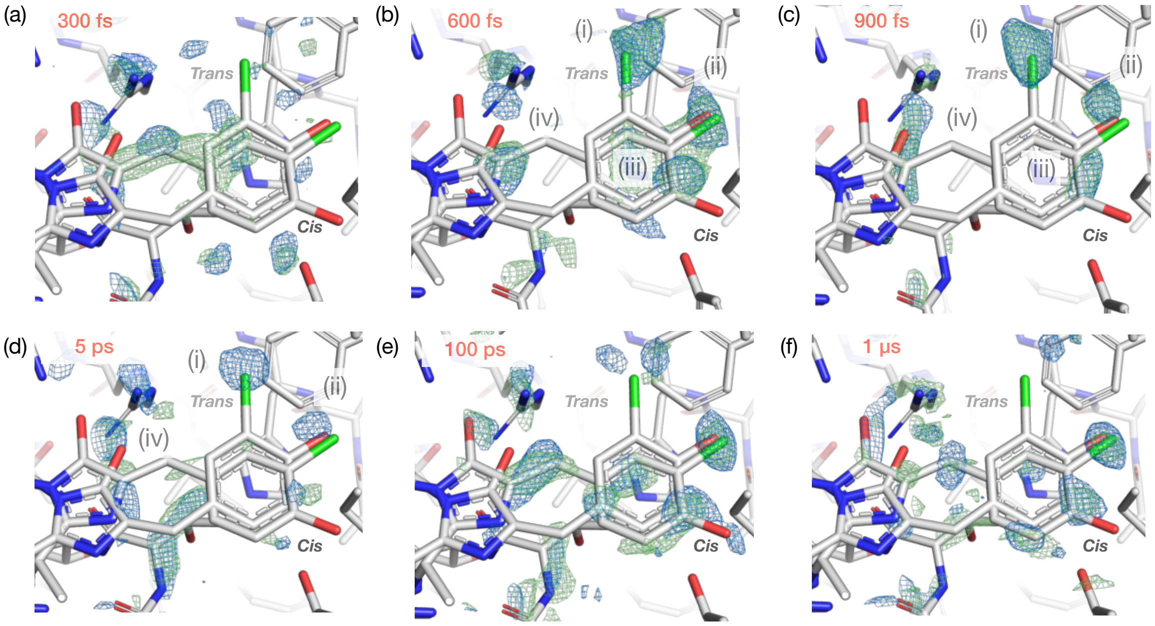

Figure S12. WΔF$_{\mathrm{max}}$ maps generated by subtraction of dark and light 2mFo-DFc maps, as described in Pearce *et al.*[1] (in green), overlaid with the WΔF$_{\mathrm{max}}$ maps from Figure S11 (in blue), both shown at +2.1σ. The two sets of maps largely overlap. The following four characteristics, which we attribute to the presence of a femtosecond intermediate, are marked in the 600 fs and 900 fs maps: (i) the presence of Peak 1 (ii) an elongated and uncentered peak where the *cis anti* chlorine is positioned, which is in contrast with the round, centered features visible in the WΔF$_{\mathrm{max}}$ maps from the later time points (iii) electron density that fills the *cis anti* chromophore phenol ring (iv) features that suggest a tilt of the imidazolidone ring oxygen towards the phenol ring. Related to Figures 4 and 5.

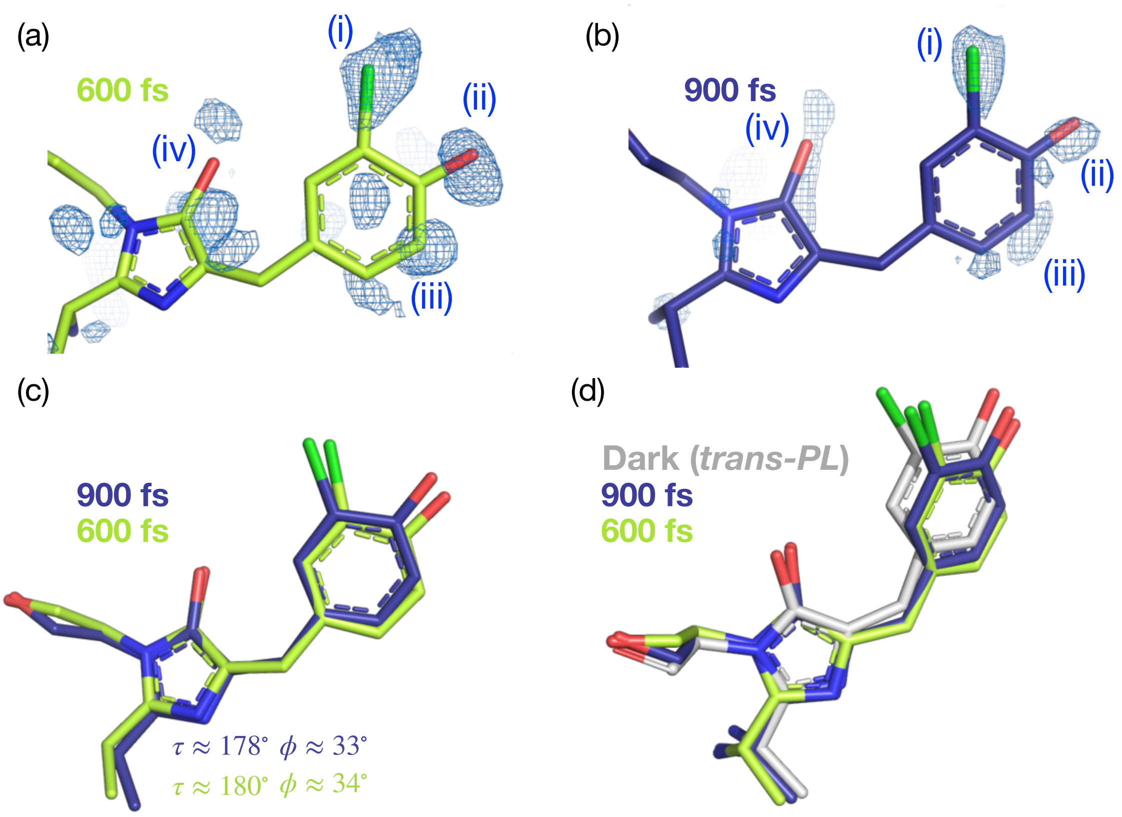

Figure S13. The WΔF$_{\mathrm{max}}$ maps for the **(a)** 600 fs and **(b)** 900 fs datasets are shown together at +2.1σ with the respective refined chromophore coordinates. The following four characteristics of these maps are marked: (i) the presence of Peak 1 (ii) a peak where the phenol ring oxygen is positioned by refinement (iii) electron density for two further carbons of the phenol ring (iv) features that suggest a tilt of the imidazolidone ring oxygen towards the phenol ring. **(c-d)** show the superposition of the two femtosecond refined structures, which we attribute to the intermediate *trans-FS*, and their relationship to the dark *trans-PL* configuration. Related to Figures 4 and 5.

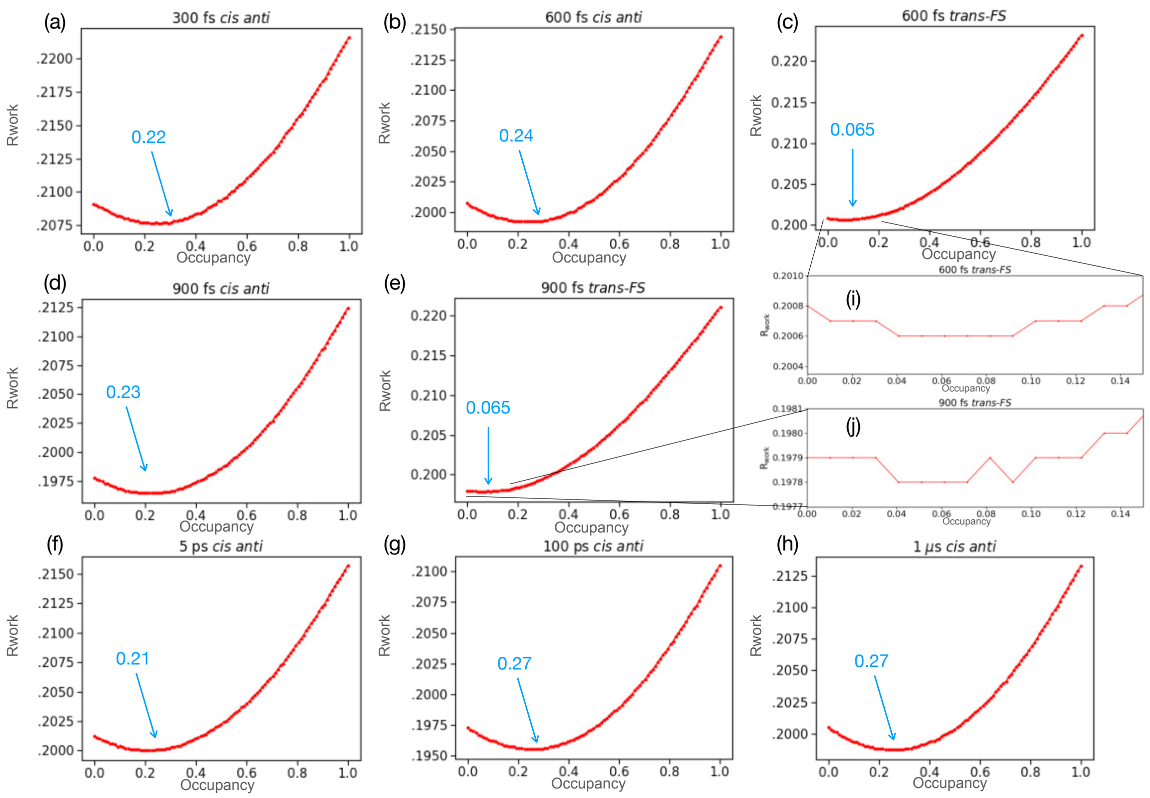

Figure S14. Occupancy refinement for light-induced chromophore conformations. These values were used to establish occupancies for the light coordinates (in order: PDBs 8A6N, 8A6O, 8A6P, 8A6Q, 8A6R, 8A6S). Related to Figure 4.

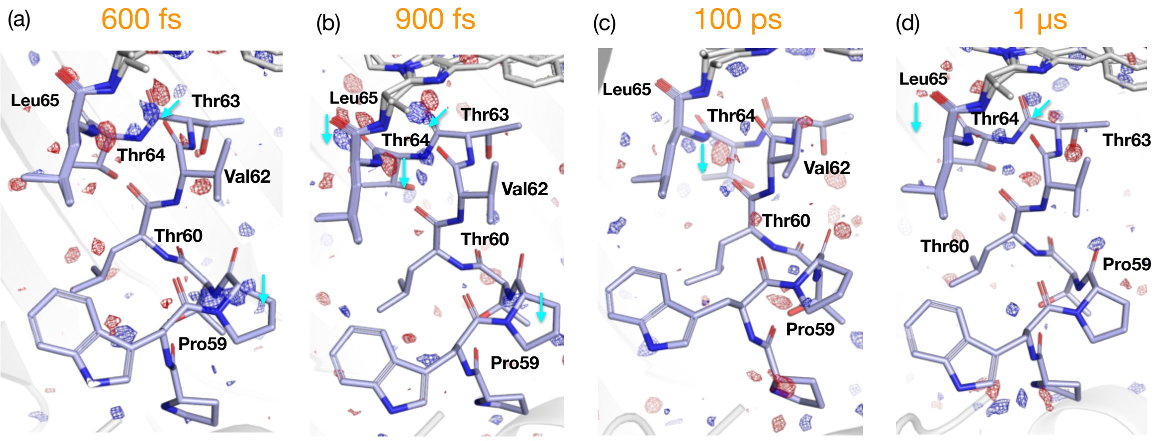

Figure S15. Difference electron density (DED) signals in the region of the central α-helix. WΔF_{max} maps are shown for each timepoint at +/-3σ (red=negative, blue=positive). The labeled residues present the most significant DED features and suggest an overall downward movement of the helix on a sub-picosecond timescale, which is particularly evident looking at residues Leu65, Thr63, and Pro59. Similar signals persist at the longer time points **(c-d)** but are weaker. Related to Figure 5.

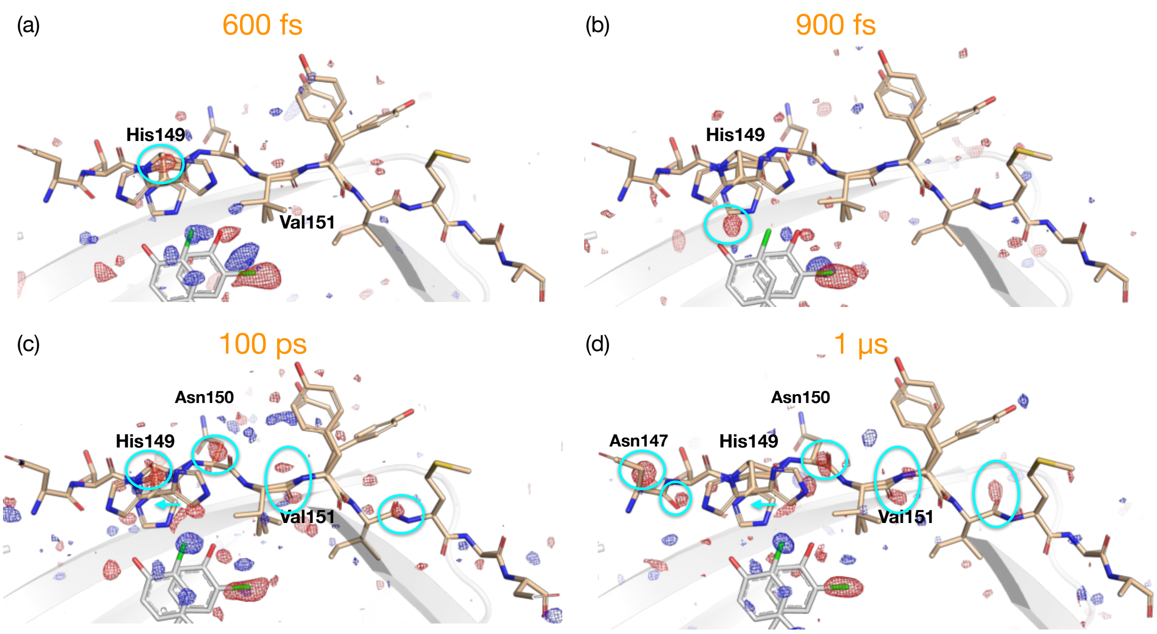

Figure S16. Difference electron density (DED) signals in the region of the β7 sheet. WΔF_{max} maps are shown for each timepoint at +/-3σ (red=negative, blue=positive). The femtosecond maps **(a-b)** have strong negative features on one of the His149 conformations. At 100 ps and 1 μs **(c-d)**, there are negative signals on the side chains and backbone of residues of Asn147, Asn150, and Val151. These are superimposable between the two datasets and suggest a downward movement of the *beta*-sheet away from the chromophore. The His149 flip that accompanies the *trans-to-cis* isomerization of the chromophore is marked by negative density on the *trans-PL*-associated conformation of this residue. Related to Figure 5.

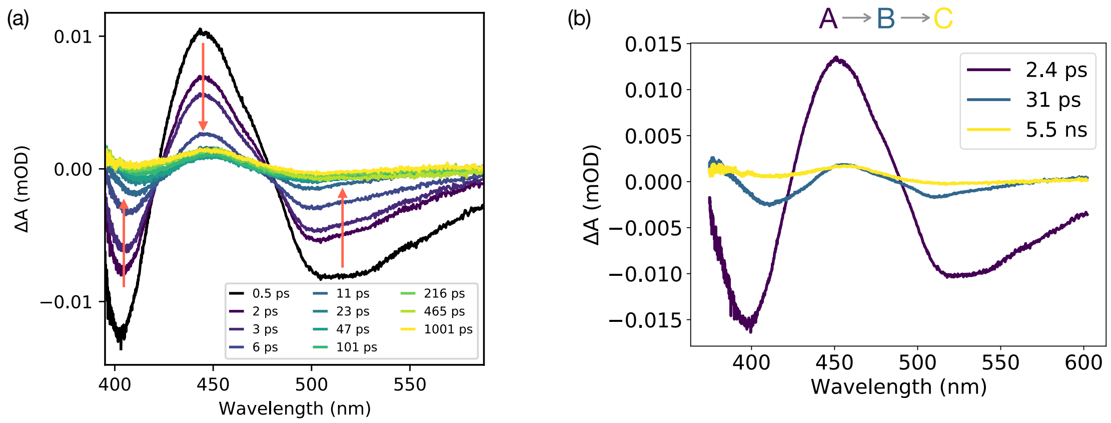

Figure S17. **(a)** Transient difference absorption spectra recorded at different pump-probe time delays after a femtosecond laser excitation (400 nm) starting from the rsEGFP2 (non-chlorinated) OFF state. **(b)** Components fitted through global analysis of the data shown in **(a)**, using a sequential model. Related to Figure 6.

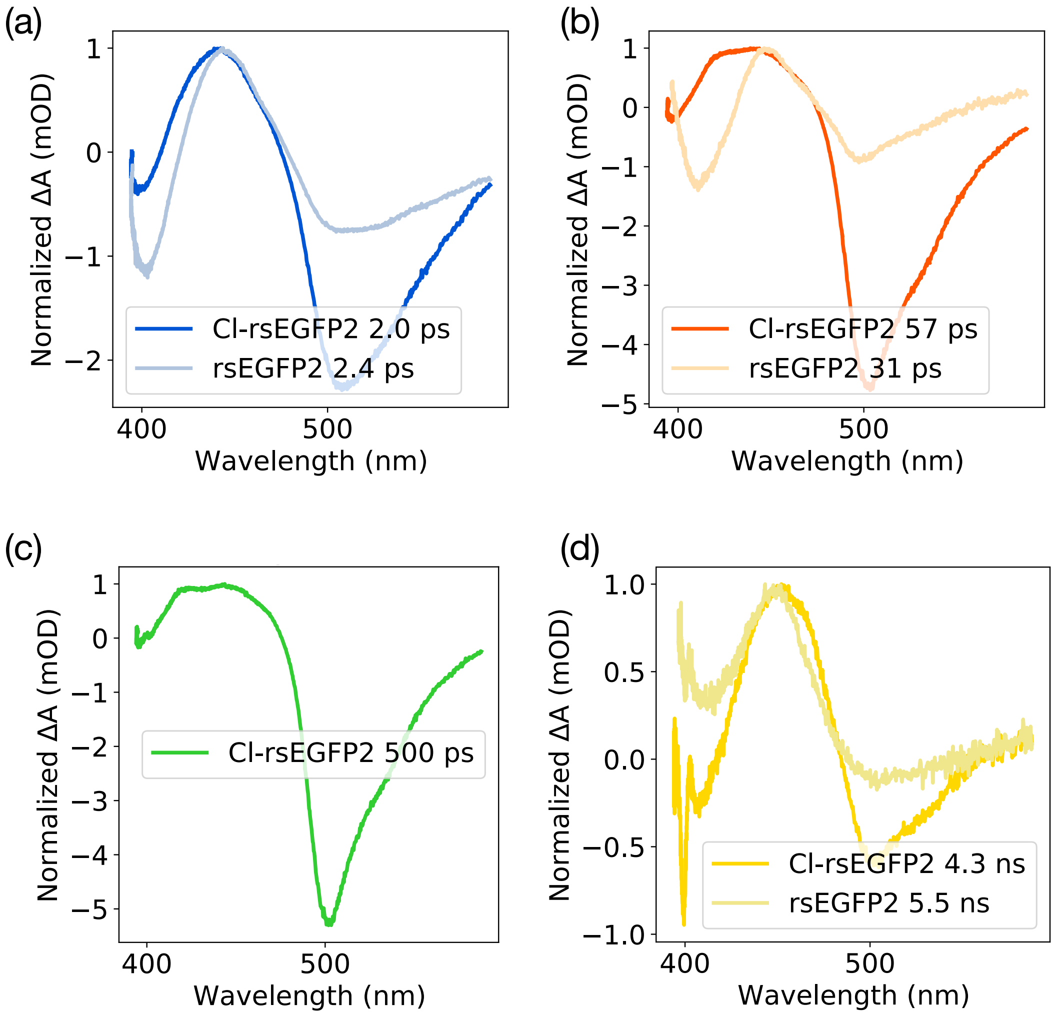

Figure S18. UV-Vis TA global analysis results comparison for rsEGFP2 (non-chlorinated) and Cl-rsEGFP2. Respective components are overlapped in **(a-d)**. Related to Figure 6.

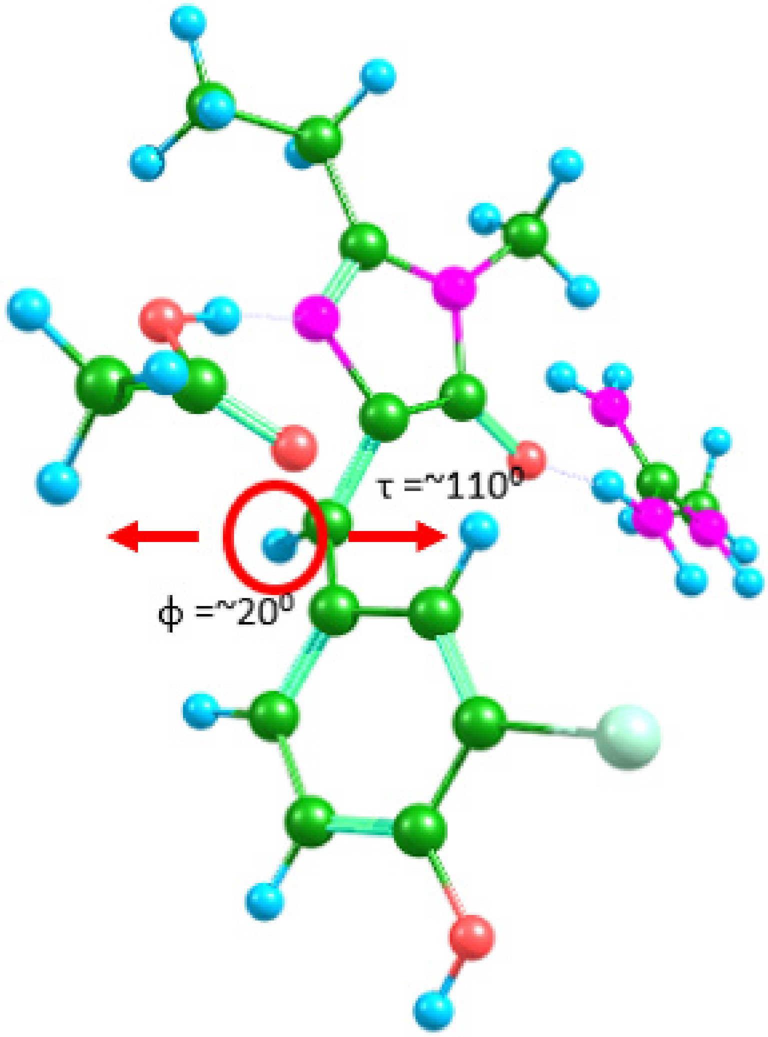

Figure S19. Geometry of the QM atoms at minimum energy conical intersection point (MECI) optimized with QM-MM. Red arrows indicate movement of the bridge hydrogen required to reach *trans* (left arrow) or *cis* (right arrow) isomers of the chromophore upon deactivation. Related to Figure 7.

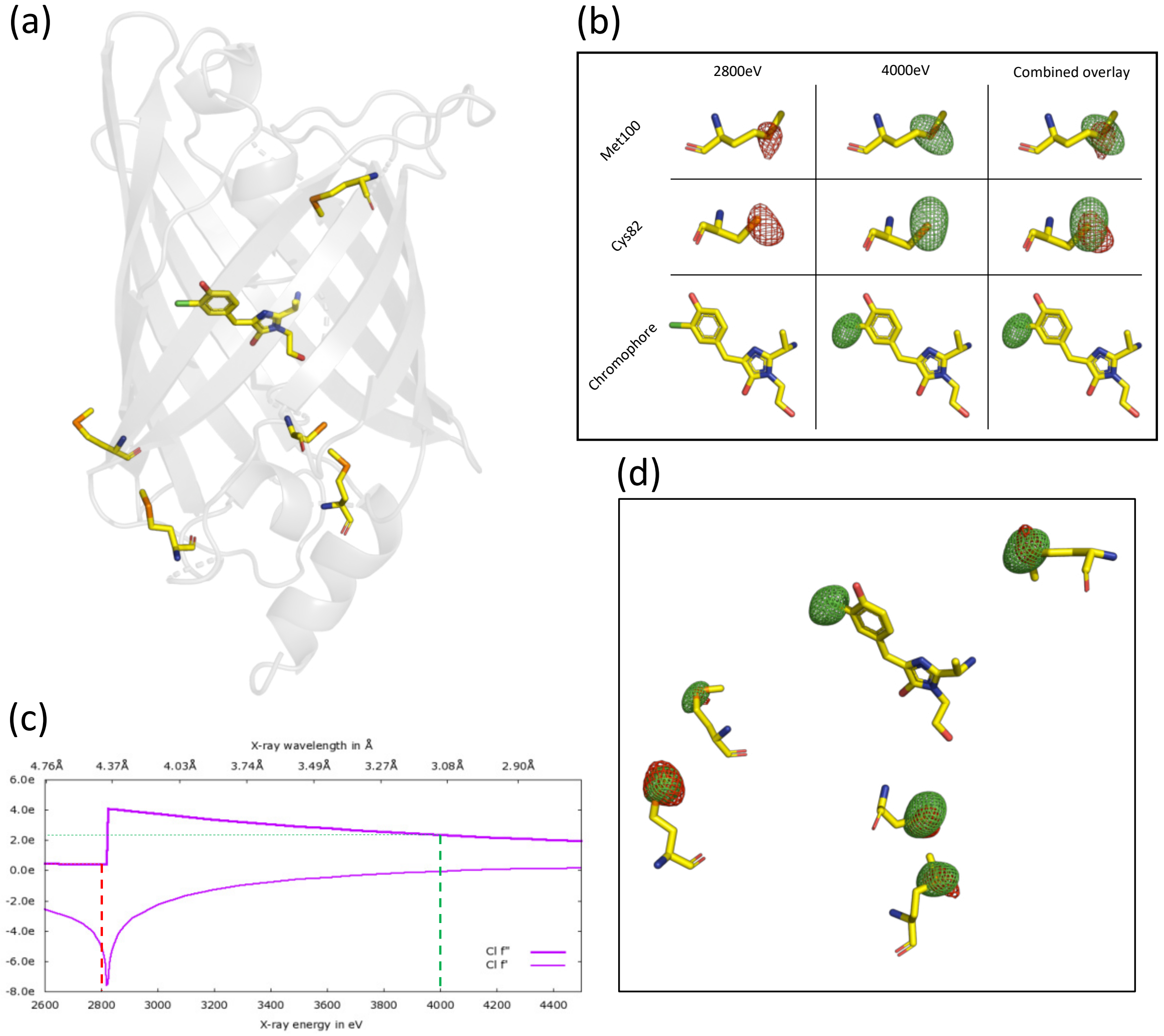

Figure S20. (a) Cartoon overview of Cl-rsEGFP2 with anomalous scatterer-containing residues and chromophore locations shown as sticks. **(b)** Anomalous difference Fourier maps for Met100, Cys82 and the chromophore above (4000 eV) and below (2800 eV) the chlorine K edge. Density presence in both maps indicates a sulphur atom, presence in the 4000 eV map combined with absence in the 2800 eV map confirms chlorine location. **(c)** f' and f" plot for Cl over the wavelength range used. Data collection wavelengths indicated as dashed lines, green for 4000eV above chlorine K edge, red for 2800 eV below. **(d)** Anomalous difference Fourier maps, both at 2800 eV and 4000 eV, for all scatterers in the structure shown with respective residues and chromophore. Related to Figure 3.

Name:	dark_CW-400nm-scale.hkl	neg5ps-400nm-scale.hkl	300fs-400nm-scale.hkl	600fs-400nm-scale.hkl	900fs-400nm-scale.hkl	5ps-400nm-scale.hkl	100ps-400nm-scale.hkl	1us-400nm-scale.hkl
Indexed Patterns:	56163	48496	18634	37132	37782	29251	55733	40977
Resolution Limits (Å):	31.46-1.80 (1.864-1.800) (1)	31.46-1.80 (1.864-1.800) (1)	31.46-1.80 (1.864-1.800) (1)	31.46-1.80 (1.864-1.800) (1)	31.46-1.80 (1.864-1.800) (1)	31.46-1.80 (1.864-1.800) (1)	31.46-1.80 (1.864-1.800) (1)	31.46-1.80 (1.864-1.800) (1)
	31.46-1.70 (1.761-1.700) (2)	31.46-1.70 (1.761-1.700) (2)	31.46-1.70 (1.761-1.700) (2)	31.46-1.70 (1.761-1.700) (2)	31.46-1.70 (1.761-1.700) (2)	31.46-1.70 (1.761-1.700) (2)	31.46-1.70 (1.761-1.700) (2)	31.46-1.70 (1.761-1.700) (2)
	31.46-1.60 (1.657-1.600) (3)	31.46-1.60 (1.657-1.600) (3)	31.46-1.60 (1.657-1.600) (3)	31.46-1.60 (1.657-1.600) (3)	31.46-1.60 (1.657-1.600) (3)	31.46-1.60 (1.657-1.600) (3)	31.46-1.60 (1.657-1.600) (3)	31.46-1.60 (1.657-1.600) (3)
No. Unique reflection Indices:	22565 (1)	22565 (1)	22565 (1)	22565 (1)	22565 (1)	22565 (1)	22565 (1)	22565 (1)
	26688 (2)	26688 (2)	26688 (2)	26688 (2)	26688 (2)	26688 (2)	26688 (2)	26688 (2)
	31903 (3)	31903 (3)	31903 (3)	31903 (3)	31903 (3)	31903 (3)	31903 (3)	31903 (3)
No. Merged Reflections:	16501938 (932123) (1)	14362196 (812915) (1)	5563788 (311307) (1)	10768669 (599450) (1)	11443185 (642832) (1)	8984192 (518649) (1)	15876885 (886790) (1)	11405674 (620880) (1)
	17947205 (877345) (2)	15629890 (770468) (2)	6043619 (290868) (2)	11695184 (562109) (2)	12441190 (605769) (2)	9801052 (497179) (2)	17244054 (829337) (2)	12351835 (570486) (2)
	19272677 (726507) (3)	16793655 (638406) (3)	6477970 (238031) (3)	12533266 (457868) (3)	13354810 (500733) (3)	10567486 (424206) (3)	18479758 (674892) (3)	13179653 (448944) (3)
Completeness (%):	100.00 (100.00) (1)	100.00 (100.00) (1)	100.00 (100.00) (1)	100.00 (100.00) (1)	100.00 (100.00) (1)	100.00 (100.00) (1)	100.00 (100.00) (1)	100.00 (100.00) (1)
	100.00 (100.00) (2)	100.00 (100.00) (2)	100.00 (100.00) (2)	100.00 (100.00) (2)	100.00 (100.00) (2)	100.00 (100.00) (2)	100.00 (100.00) (2)	100.00 (100.00) (2)
	100.00 (100.00) (3)	100.00 (100.00) (3)	100.00 (99.97) (3)	100.00 (100.00) (3)	100.00 (100.00) (3)	100.00 (100.00) (3)	100.00 (100.00) (3)	100.00 (100.00) (3)
Signal to noise:	9.007 (3.79) (1)	8.395 (3.54) (1)	5.239 (2.18) (1)	7.329 (3.08) (1)	7.442 (3.24) (1)	6.753 (3.05) (1)	9.080 (3.71) (1)	7.682 (3.11) (1)
	8.028 (2.44) (2)	7.481 (2.28) (2)	4.664 (1.38) (2)	6.526 (1.97) (2)	6.647 (2.11) (2)	6.048 (2.01) (2)	8.071 (2.32) (2)	6.821 (1.89) (2)
	6.973 (1.36) (3)	6.500 (1.30) (3)	4.052 (0.79) (3)	5.661 (1.08) (3)	5.787 (1.23) (3)	5.278 (1.21) (3)	6.987 (1.26) (3)	5.903 (1.04) (3)
Wilson b factor:	27.58 (1)	27.41 (1)	27.79 (1)	27.48 (1)	27.44 (1)	27.22 (1)	27.79 (1)	28.00 (1)
	29.27 (2)	29.23 (2)	29.57 (2)	29.25 (2)	29.12 (2)	28.93 (2)	29.57 (2)	29.82 (2)
	30.19 (3)	30.11 (3)	30.29 (3)	30.27 (3)	29.99 (3)	29.85 (3)	30.56 (3)	30.57 (3)
R_{Split} (%):	8.89 (30.14) (1)	9.98 (32.17) (1)	15.55 (53.80) (1)	11.34 (37.64) (1)	10.64 (34.53) (1)	12.22 (36.28) (1)	8.71 (32.23) (1)	10.45 (37.26) (1)
	9.11 (51.23) (2)	10.21 (54.62) (2)	15.95 (94.06) (2)	11.62 (63.96) (2)	10.90 (60.53) (2)	12.49 (62.56) (2)	8.95 (54.93) (2)	10.72 (67.38) (2)
	9.40 (101.17) (3)	10.53 (106.51) (3)	16.50 (183.78) (3)	11.99 (132.67) (3)	11.26 (116.74) (3)	12.85 (120.72) (3)	9.26 (112.17) (3)	11.11 (139.69) (3)
CC*	1.00 (0.97) (1)	1.00 (0.97) (1)	0.99 (0.92) (1)	0.99 (0.96) (1)	1.00 (0.97) (1)	0.99 (0.96) (1)	1.00 (0.97) (1)	0.99 (0.96) (1)
	1.00 (0.94) (2)	1.00 (0.93) (2)	0.99 (0.84) (2)	0.99 (0.90) (2)	1.00 (0.92) (2)	0.99 (0.90) (2)	1.00 (0.93) (2)	0.99 (0.91) (2)
	1.00 (0.83) (3)	1.00 (0.81) (3)	0.99 (0.59) (3)	0.99 (0.74) (3)	1.00 (0.77) (3)	0.99 (0.77) (3)	1.00 (0.81) (3)	1.00 (0.74) (3)
$CC_{1/2}$	0.99 (0.90) (1)	0.98 (0.90) (1)	0.96 (0.74) (1)	0.98 (0.86) (1)	0.98 (0.88) (1)	0.98 (0.85) (1)	0.99 (0.89) (1)	0.98 (0.86) (1)
	0.99 (0.79) (2)	0.98 (0.76) (2)	0.96 (0.55) (2)	0.98 (0.69) (2)	0.98 (0.72) (2)	0.98 (0.69) (2)	0.99 (0.76) (2)	0.98 (0.71) (2)
	0.99 (0.52) (3)	0.98 (0.48) (3)	0.96 (0.21) (3)	0.98 (0.38) (3)	0.98 (0.43) (3)	0.98 (0.42) (3)	0.99 (0.48) (3)	0.98 (0.38) (3)

Table S1. CrystFEEL merging statistics for the eight datasets collected, reported at three different high resolution cutoffs.

Dataset :	SACLA 2021 -5ps Cl-rsEGFP2	SACLA 2021 Interleaved Dark Cl-rsEGFP2	SACLA 2019 Interleaved Dark rsEGFP2	SSRL Cryo + Dehydrated Cl-rsEGFP2
Resolution range :	1.63 (1.688 - 1.63)	1.63 (1.688 - 1.63)	19.1 - 1.463 (1.515 - 1.46)	38.79 - 1.81 (1.875 - 1.81)
Space group :	P 21 21 21	P 21 21 21	P 21 21 21	P 21 21 21
Unit cell :	51.99 62.91 72.03 90 90 90	51.99 62.91 72.03 90 90 90	51.97 63.04 72.03 90 90 90	51.48 59.00 64.48 90 90 90
Reflections used in refinement :	30141 (2952)	30141 (2951)	36821 (340)	18407 (1804)
Reflections used for R-free :	1510 (146)	1510 (146)	1790 (19)	921 (91)
R-work :	0.1685 (0.3102)	0.1634 (0.2939)	0.1696 (0.4389)	0.2027 (0.3068)
R-free :	0.2013 (0.3369)	0.2000 (0.3428)	0.1952 (0.4783)	0.2266 (0.3376)
Number of non-hydrogen atoms :	2277	2277	2097	1906
macromolecules	2089	2089	1883	1756
ligands	63	63	40	41
solvent	125	125	174	109
Protein residues :	235	235	235	225
RMS(bonds) :	0.017	0.017	0.027	0.007
RMS(angles) :	1.46	1.46	1.78	0.93
Ramachandran favored (%) :	99.13	99.13	99.13	98.64
Ramachandran allowed (%) :	0.87	0.87	0.87	1.36
Ramachandran outliers (%) :	0	0	0	0
Rotamer outliers (%) :	2.09	2.09	1.9	0
Clashscore :	6.16	6.16	3.17	2.01
Average B-factor :	28.44	28.44	27.37	34.47
macromolecules	28.23	28.23	26.4	34.26
ligands	19.27	19.27	16.28	35.01
solvent	36.56	36.56	40.46	37.7

Table S2. Refinement statistics for: the refined dark model from the SACLA 2021 experiment computed using the interleaved dark (dark-CW) and negative 5 ps datasets, the refined dark model from the SACLA 2019 experiment, the SSRL cryo-dataset. Related to Figures 3-5.

Structure	Construct	Main *trans* Configuration	Method	Unit Cell (a b c – Å)
**6PFT**	Cl-rsEGFP2	*trans-TW* (*syn*)	irradiate -> cryocool	51.0 62.7 68.6
**6PFU**	Cl-rsEGFP2	*trans-PL* (*anti*)	dehydrate -> irradiate -> cryocool	51.3 59.5 65.4
8A83	Cl-rsEGFP2	*trans-PL* (*anti*)	irradiate -> dehydrate -> cryocool	51.5 59.0 64.5
8A6G	Cl-rsEGFP2	*trans-PL*	dark, room temperature SFX data	52.0 63.0 72.0
8A7V	rsEGFP2	*trans-TW*	dark, room temperature SFX data	52.0 63.0 72.0
7O7X	rsEGFP2 V151A	*trans-TW*	dark, room temperature SFX data	51.8 62.7 71.6
7O7W	rsEGFP2 V151L	*trans-PL*	dark, room temperature SFX data	51.8 62.9 71.9

Table S3. Summary table of unit cell dimensions for rsEGFP2 structures (chlorinated and non-) from this and other studies. Related to Figure 3.

Supporting Notes

1. OFF State SFX Structure for Cl-rsEGFP2

Our room temperature dark structure for Cl-rsEGFP2 presents predominantly the planar *trans anti* configuration (*trans-PL*) and minor populations of *trans syn* (*trans-TW*), and *cis anti* configurations (Figure S4). A previous cryotrapping crystallographic study[2] identified the *trans-PL* and *trans-TW* configurations as photoproducts of the HT and OBF pathways respectively. The *trans-PL* photoproduct was found in the structure with a contracted unit cell, while *trans-TW* was the primary configuration in the structure from the larger unit cell. This led to the conclusion that the choice of pathway is dependent on the crystal packing, where tighter packing favors the volume-conserving HT. Interestingly, our dark room temperature SFX structure presents mainly the *trans-PL* form, despite its large unit cell, when the chlorine substituent is present (Table S3 and Figure S5). This suggests that the *cis*-to-*trans* isomerization pathway at room temperature is a hula-twist, independent of unit cell dimensions. Table S3 lists the predominant conformations for the Cl-rsEGFP2 structures in this paper and in the cryotrapping study[2], with the corresponding treatments, as well as other published rsEGFP2 SFX structures. Room temperature SFX data indicate that the difference in chromophore conformation observed by Chang *et al.*[2] was induced by the dehydration protocol or the freezing process itself, rather than dictated by a lattice-dependent change in pathway. This interpretation is further supported by a separate cryotrapping experiment (row 3 of Table S3 and Figure S6, PDB 8A83) where the ON state crystal is first irradiated with 488 nm light, dehydrated into the smaller unit cell size, and then subsequently cryocooled; the resulting structure shows a *trans-PL* chromophore.

Supporting Procedures

1. 2021 SFX Data Analysis

1.1. Frame Data Processing and Crystallographic Analysis

A bad/damaged pixel mask for the detector was generated from dark scans recorded before data collection and was applied across all image data processing. Peak and hit finding were performed using Cheetah[3,4]. Identified hits were then indexed and integrated in CrystFEL[5,6] using the XGANDALF algorithm[7]. The achieved indexing rate was around 80% for all data. Indexed crystals from CrystFEL streamfiles were binned into different time delays and separated as "light" (pump laser on) and "dark" (pump laser off) to generate respective reflection files using a custom python script. Merging statistics for the datasets collected are reported in Table S1. The PHENIX[8] reflection conversion function was used to convert intensities to structure factors and model refinement was done using the phenix.refine function. Minor chromophore

populations in the dark structure were manually refined (PDB 8A6G). Low resolution (30 Å) and high resolution (1.63 Å) cutoffs were applied. Q-weighted difference electron density maps and background subtracted maps were then generated as explained in Section 2 of the Supporting Procedures.

For the analysis of protein-wide pump-induced changes, we used the scripts by Wickstrand *et al.*[9]. In short, a spherical volume of radius 2.0 Å was walked across every atom in the protein and the positive and negative difference electron densities from each dataset's Q-weighted map were averaged separately within each volume. A cut-off of +/-3σs and a grid spacing of 0.5 Å were used in the scripts.

1.2. Time Zero Determination by Cross Correlation

The cross-correlation of the optical and X-rays pulses was measured via the standard technique, previously described[10]. Briefly, a 50 μm thick, semi-conducting crystal (Ce:YAG) was placed in the interaction region. The transmission intensity of the optical pulse through the crystal was measured by a photo-diode. Exposure to hard X-rays causes modulation of the crystal's reflective and transmissive properties. The amplitude of the optical transmission was monitored while the temporal delay between the two pulses was scanned. With appropriate volume of data and averaging, the resulting signal exhibits a step-like function, centred approximately when the two pulse are overlapped in the temporal delay. The signal measured on the photo-diode (Figure S3(a)) as a function of delay was fitted as previously described[11]. The averaged time zero over a number of runs was used to correct the delay stage, to give an accurate time zero and, as such, an accurate binning for each data set (300 fs, 600 fs, 900 fs, 5 ps).

SACLA has recently implemented a feedback system between the optical and X-ray pulse, using a balanced optical-microwave phase detector (BOMPD)[12]. This corrects for long-term temporal drift over several hours or days which previously caused movement on the order of 60 - 100 fs[11]. The BOMPD feedback loop, additionally, reduces X-ray jitter stemming from an intrinsic instability in the Self Amplified Spontaneous Emission (SASE) process to sub 50 fs. Since it was difficult to suppress the actual timing drift using only a BOMPD, the drift here was further compensated using a phase shifter and the timing tool data. The jitter measured from the cross-correlation fittings is shown in (Figure S3(b)). As an additional precaution to mitigate drift, the cross-correlation was measured before the start of each day. Furthermore, the reflection intensity (transmission^{-1}) was measured and used as a comparative cross-correlation.

With the various steps taken described above, as-well as the new feedback system implemented at SACLA, we were able to accurately bin the data into sub picosecond bins (300, 600 and 900 fs) and confidently ascribe the ultrafast dynamics.

1.3. Generation of Q-weighted Maps

Through the *reciprocalspaceship* library[13], dark and light structure factors (F_{obs_D} and F_{obs_L} respectively) are in turn scaled to the structure factors calculated from the refined dark structure (F_{calc}).A simple scale factor *m* is applied to an entire dataset (Figure S7(a-b)). Q-weighted difference electron density maps are then calculated using weighted structure factor amplitudes, where the Bayesian weight applied is based on the work of Ursby *et al.*[14,15] and implemented in python using *reciprocalspaceship* (Figure S7(c-d)). A value of α = 0.2 is used here. Maps where the scaling is done using the SCALEIT program within the CCP4 suite[16] and the Q-weighting is implemented as in[17–19] present the same key features as those generated by the method described here (not shown). The easy implementation in python, however, allows for easy manipulation and visualization of the structure factor distributions and screening of improved map generation parameters (such as scale factor and α values).

1.4. Principal Component Analysis (PCA) of Electron Density Maps

Principal component analysis (PCA) of the Q-weighted maps described above is performed in python by loading each map as a NumPy array through the GEMMI library for structural biology (GEMMI version 0.4.5). A solvent mask and a mask that only retains grid points within 7 Å of the chromophore are applied. PCA is then sklearn.decomposition.PCA function (Scikit-learn version 1.0.1). Figure S8 presents the pipeline and results for PCA on the six DED maps. The first 5 components (C1-C5) are shown (noting that the

component sign is arbitrary in the analysis). PCA here is used primarily as a method to improve the data signal-to-noise ratio: the 6th component (C6) is identified as noise and the final difference maps shown in Figure 4 are reconstructed without it.

1.5. Generation of Background Subtracted Maps (WΔF_{max} maps)

In order to refine light-induced coordinates, one ideally wants to separate the signal that belongs to the photoproduct population from that of the dark state. Because only a small percentage of protein molecules actually undergo photoactivation, the problem of extracting the electron density of the photoproduct is a very similar issue to the one Pearce and colleagues describe of discerning minor states in macromolecular crystallography[1]. Pearce *et al.* generate background corrected maps by subtracting the "ground state" $2mF_o$-DF_c map from the "dataset" $2mF_o$-DF_c map as follows :

$$[\text{corrected map}] = [\text{dataset map}] - N_{bg} \times [\text{ground state map}] \quad (1)$$

where the background correction factor (we refer to it here as N_{bg}, in[1] it is called BDC – Background Density Correction factor) is the value that maximizes the difference in correlation (calculated from the ground state and the corrected map) between the entire protein and a specific area of change.
We have extended this here to Q-weighted maps. Figure S9 describes the steps involved in generating what we have called WΔF_{max} maps. Q-weighted maps of the form :

$$W\Delta F_{corr} = w \times (|\ F_{obs_L}\ | - N_{bg} \times |\ F_{obs_D}\ |) \quad (2)$$

are generated for a range of N_{bg} between 0 and 1 and saved as electron density maps in CCP4 format. In real space, a local region, R_{loc}, is defined as a sphere of 5 Å centered around the chromophore double bond. The entire protein is defined as R_{glob} after a solvent mask is applied. For each value of N_{bg}, we compute the Pearson correlation coefficient between the respective $W\Delta F_{corr}$ map and the map obtained from the dark model calculated structure factors (F_{calc}). This is done for both R_{loc} and R_{glob}. We then choose the N_{bg} value that maximizes the difference between these two correlation coefficients and save the corresponding map (WΔF_{max}) for that timepoint. The determination of the appropriate background subtraction value for each timepoint is shown in Figure S10 and the corresponding WΔF_{max} maps are shown in Figure S11. The *cis anti* photoproduct is discernible already at 300 fs and is very clear in the 100 ps and 1 μs maps. The maps from the 600 fs and 900 fs datasets contain additional features (numbered (i-iv)) that support the presence of the femtosecond intermediate *trans-FS* (see main text and Figure S13). The 5 ps WΔF_{max} map, just as the respective Q-weighted map, is noisier than the other datasets, though it still contains weak *trans-FS* features and some *cis anti* population. Figure S12 displays background corrected maps generated by subtraction of light and dark $2mF_o$-DF_c as done in[1]. These are very similar to the ones calculated directly from reciprocal space and support the conclusions drawn above.

Refinement of Cl-rsEGFP2 coordinates to WΔF_{max} maps is done, starting from the dark coordinates, with phenix.refine by allowing only atom positions from the chromophore and the residues immediately next to it in the sequence (residues 63-65 and 69-71) to vary. This is because the background subtracted structure factors are much lower signal-to-noise than the ones used to refine the dark structure, and we therefore limit their use to the chromophore region where light-induced changes are strong enough to clearly reveal the presence of minor populations. Occupancies for the *cis anti* and *trans-FS* species in light datasets were refined so as to minimize R_{work} (Figure S14). Despite the weak presence of *trans-FS* features in the 5 ps maps, *trans-FS* occupancy refinement (not shown) for this timepoint did not yield a significant population, so this conformation was not included in the final coordinates. Final structures for each time point are deposited (in order from 300 fs to 1 μs) as PDBs 8A6N, 8A6O, 8A6P, 8A6Q, 8A6R, 8A6S.

2. 2019 SFX for OFF-state rsEGFP2 structure

Dark data was collected at SACLA at the BL3 EH2 beamline with setup equivalent to the one described above (MPCCD-phase III detector, 10.5 keV), but a refined detector distance of 50.6 mm. Data was then

processed as described for the 2021 SFX experiment, with the difference that structure refinement was done in REFMAC[20] (PDB 8A7V).

3. Extra-Cryotrapping Experiment

Crystals were grown as described in[2]. For the irradiation/dehydration protocol, the crystal was fished onto a loop and transferred to a separate droplet of mother liquor on a cover slip. The entire cover slip was picked up with tweezers and placed in the beam of a 488nm laser (specifics as in[2]). Illumination lasted between 20-30 seconds. Following this, the crystal was transferred to a drop of dehydrating cryoprotectant[2] and placed long enough to contract into the smaller unit cell size (approximately 5 seconds). It was then fished out onto a loop again and plunged into liquid nitrogen. Data was collected at the SSRL 7-1 beamline. Data reduction and refinement were then done using XDS[21] and Phenix[8] (PDB 8A83).Refinement statistics for this cryotrapped structure and for the dark structures collected at SACLA are reported in Table S2.

4. Quantum Chemical Modeling Details

QM-MM simulations in the protein were performed using our own version of GROMACS 4.6.5[22,23] coupled to the Terachem[24,25] (for ground state) and GAMESS(US)[26] (for excited-state) quantum chemistry packages. We searched for minimum-energy geometries in all systems using the limited-memory Broyden-Fletcher-Goldfard-Shannon (L-BFGS) quasi-Newton optimization algorithm, without PBC and with infinite cut-offs for the Coulomb and Lennard-Jones interactions. All optimizations in the work were performed until the maximum component of the force was lower than 10 kJ/mol/nm. Vertical absorption and emission spectra calculations were performed with xMCQDPT2 method implemented in the Firefly package[27].

4.1. Planar Ground and Excited State trans-PL Structures

Initially, we performed optimization of the Cl-rsEGFP2 on ground and excited states, starting from the dark *trans-PL* crystal structure. On the ground state, we performed optimization with density functional theory (DFT) at PBE0/cc-pVDZ//Amber03[28–30] with empirical corrections to dispersion energies and interactions introduced with Grimme's DFT-D3 model[31]. On the excited state, a double optimization scheme was employed. At first, SA2-CASSCF(2,2)/3-21G//Amber03[32] was used to minimize the S1 state. We used a small active space in this optimization to prevent the interchange between the S1 and S2 states, which typically happens for the neutral GFP chromophores when using using larger active spaces without electron correlation. The structure optimised with the small active space, was subject to a second optimisation at the SA2-CASSCF(12,11)/3-21G//Amber03 level of theory.

Both S0 and S1 optimized structures are planar. Next, we computed vertical excitation and emission energies at the xMCQDPT2/SA6-CASSCF(12,11)/cc-pVDZ//Amber03 level of theory. All 6 states were included into averaging as well as into the effective Hamiltonian. Results suggest absorption at 405 nm (3.06 eV) and emission at 518 nm (2.39 eV). In addition for the S1 minimum energy geometry, we computed an excited-state absorption (ESA) from the S1 into the S5 of 425 nm (2.92 eV). These excitation energies are in line with the experimental data (Figure 6), and hence suggest that the model provides an adequate qualitative description for our system.

4.2. Identification of the Photoisomerization Pathway

At first, we performed a search for the isomerization pathway connected to the TR-SFX-resolved *trans-FS* structure at 900 fs. Optimization on the ground (PBE0/cc-pVDZ//Amber03) and excited (SA2-CASSCF(12,11)/3-21G//Amber03) states resulted in the planar *trans-PL* structure, suggesting that this twisted structure is not connected to any minima on the excited state. To identify minimum energy conical intersection points(MECI), we implemented a penalty function MECI search algorithm[33] with α=0.02 and σ=16 as the parameters for the penalty function. When starting the optimization from the the *trans-FS* structure, no MECI could be located, in line with previous computational studies[34,35] that suggest that in the neutral GFP-chromophores there is no conical intersection associated with the rotation of the phenol ring.

We next searched for a hula-twist MECI by manually increasing τ torsion angle by 45° in the *trans-FS* structure and optimising its geometry at the SA2-CASSCF(12,11)/3-21G//Amber03 level of theory. This optimisation resulted in a twisted structure with an S1/S0 energy gap of ≈0.035 a.u. Starting a MECI optimization from this geometry leads to the MECI structure shown in Figure S19 with an S1/S0 energy gap of ≈0.0005 a.u. After the optimizations we also recomputed energies for both twisted minima and MECI points with xMCQDPT2/SA6-CASSCF(12,11)/cc-pVDZ//Amber03. In addition, to determine whether the MECI point connects the anti-trans conformer to the anti-cis conformer, we performed a ground state optimisation at PBE0/cc-pVDZ//Amber03 level starting from the MECI. While a direct optimization leads to the original planar *anti-trans-PL* chromophore conformer, a slight perturbation of the position of the methylene hydrogen atom by ≈ 0.3 Å towards a more *cis*-like position, leads to the *anti-cis* conformer.

5. Confirming the Presence of the Chlorine Substituent

To confirm incorporation of the chlorine substituent into the chromophore, we analyzed anomalous difference Fourier maps from anomalous data taken at different wavelengths. Single crystals were obtained by the hanging-drop vapor diffusion method at 20 °C described previously[36]. Briefly, the protein solution (12 mg/mL, 50mM Hepes pH 7.5, 20 mM NaCl) was mixed 1:1 with the precipitant solution (100 mM Hepes pH 8.0, 1.80 M ammonium sulphate, 20 mM NaCl) to yield 2-4 muL drops that were placed over a well containing 1 mL of the precipitant solution. Mature crystals with dimensions up to 500 muLm x 200 muLm x 200 muLm appeared after three weeks. Prior to flash-freezing in liquid nitrogen, crystals were cryoprotected by passage through three solutions of incrementally increasing amounts of sucrose to a final concentration of 1.2M in 75 mM Hepes pH 8.1, 20 mM NaCl, 0.9 M ammonium sulphate.

Data was collected on beamline I23, Diamond Light Source, using the Pilatus 12M semi-cylindrical detector at a temperature of 50K[37]. Data were collected above and below the chlorine K absorption edge at 4.0 keV (λ=3.1 Å) and 2.8 keV (λ=4.4Å) respectively. Each dataset consisted of 3600 images at 0.1^{c}irc oscillation with 0.1s exposure. 3 x 360^{c}irc datasets at 4.0 keV were used to phase the structure using CRANK2. Further structure refinement was performed in Phenix and Coot (PDB 8AM4). Anomalous difference Fourier maps were generated using ANODE[38]. Figure S20 shows the resulting anomalous difference Fourier maps from 4.0 keV and 2.8 keV. The presence of density in the 4.0 keV dataset (green mesh, 5σ) in conjunction with the absence of density in the 2.8 keV dataset (red mesh, 4σ) is conclusive of Cl, confirming the identity of the chromophore heavy atom substitution.

6. OFF-to-ON Quantum Yield for the Cl-rsEGFP2 construct

We carried out a comparative measurement to estimate the reaction quantum yield (QY) for our chlorinated rsEGFP2 construct. The OFF-to-ON QY for unchlorinated rsEGFP2 was most recently estimated to 0.23[39]. rsEGFP2 and Cl-rsEGFP2 solutions were fully converted to the OFF state using through illumination with a 488 nm LED. The final OD at 405 nm for both was ≈ 0.25. The OFF-to-ON conversion was then driven with a 405 nm LED at 4.4 mW power and the absorption at 488 nm and 405 nm monitored with an Agilent 8453 spectrophotometer.

Triplicate measurements were averaged and normalized. Figure S1 shows the 405 nm absorption decay for both constructs with the respective fitted decay rates (k). From the ratio of the two fitted rates we estimate the OFF-to-ON QY for Cl-rsEGFP2:

$$\phi_{Cl} = \phi_{noCl} \times \frac{k_{Cl}}{k_{noCl}} = 0.2 \tag{3}$$

Supporting References

1. Pearce, N.M.; Krojer, T.; Bradley, A.R.; Collins, P.; Nowak, R.P.; Talon, R.; Marsden, B.D.; Kelm, S.; Shi, J.; Deane, C.M.; von Delft, F. A multi-crystal method for extracting obscured crystallographic states from conventionally uninterpretable electron density. *Nature Communications* **2017**, *8*, 15123. doi:10.1038/ncomms15123.
2. Chang, J.; Romei, M.G.; Boxer, S.G. Structural Evidence of Photoisomerization Pathways in Fluorescent Proteins. *Journal of the American Chemical Society* **2019**, *141*, 15504–15508. doi:10.1021/jacs.9b08356.
3. Barty, A.; Kirian, R.A.; Maia, F.R.N.C.; Hantke, M.; Yoon, C.H.; White, T.A.; Chapman, H. Cheetah : software for high-throughput reduction and analysis of serial femtosecond X-ray diffraction data. *Journal of Applied Crystallography* **2014**, *47*, 1118–1131. doi:10.1107/S1600576714007626.
4. Nakane, T.; Joti, Y.; Tono, K.; Yabashi, M.; Nango, E.; Iwata, S.; Ishitani, R.; Nureki, O. Data processing pipeline for serial femtosecond crystallography at SACLA. *Journal of Applied Crystallography* **2016**, *49*, 1035–1041. doi:10.1107/S1600576716005720.
5. White, T.A.; Kirian, R.A.; Martin, A.V.; Aquila, A.; Nass, K.; Barty, A.; Chapman, H.N. CrystFEL : a software suite for snapshot serial crystallography. *J. Appl. Cryst* **2012**, *45*, 335–341. doi:10.1107/S0021889812002312.
6. White, T.A.; Mariani, V.; Brehm, W.; Yefanov, O.; Barty, A.; Beyerlein, K.R.; Chervinskii, F.; Galli, L.; Gati, C.; Nakane, T.; Tolstikova, A.; Yamashita, K.; Yoon, C.H.; Diederichs, K.; Chapman, H.N. Recent developments in CrystFEL. *Journal of Applied Crystallography* **2016**, *49*, 680–689. doi:10.1107/S1600576716004751.
7. Gevorkov, Y.; Yefanov, O.; Barty, A.; White, T.A.; Mariani, V.; Brehm, W.; Tolstikova, A.; Grigat, R.R.; Chapman, H.N. XGANDALF – extended gradient descent algorithm for lattice finding. *Acta Crystallographica Section A Foundations and Advances* **2019**, *75*, 694–704. doi:10.1107/S2053273319010593.
8. Adams, P.D.; Afonine, P.V.; Bunkóczi, G.; Chen, V.B.; Davis, I.W.; Echols, N.; Headd, J.J.; Hung, L.W.; Kapral, G.J.; Grosse-Kunstleve, R.W.; McCoy, A.J.; Moriarty, N.W.; Oeffner, R.; Read, R.J.; Richardson, D.C.; Richardson, J.S.; Terwilliger, T.C.; Zwart, P.H.; IUCr. PHENIX: a comprehensive Python-based system for macromolecular structure solution. *Acta Crystallographica Section D Biological Crystallography* **2010**, *66*, 213–221. doi:10.1107/S0907444909052925.
9. Wickstrand, C.; Katona, G.; Nakane, T.; Nogly, P.; Standfuss, J.; Nango, E.; Neutze, R. A tool for visualizing protein motions in time-resolved crystallography. *Structural Dynamics* **2020**, *7*, 024701. doi:10.1063/1.5126921.
10. Gahl, C.; Azima, A.; Beye, M.; Deppe, M.; Döbrich, K.; Hasslinger, U.; Hennies, F.; Melnikov, A.; Nagasono, M.; Pietzsch, A.; Wolf, M.; Wurth, W.; Föhlisch, A. A femtosecond X-ray/optical cross-correlator. *Nature Photonics* **2008**, *2*, 165–169. doi:10.1038/nphoton.2007.298.
11. Sanchez-Gonzalez, A.; Johnson, A.S.; Fitzpatrick, A.; Hutchison, C.D.M.; Fare, C.; Cordon-Preciado, V.; Dorlhiac, G.; Ferreira, J.L.; Morgan, R.M.; Marangos, J.P.; Owada, S.; Nakane, T.; Tanaka, R.; Tono, K.; Iwata, S.; van Thor, J.J. Coincidence timing of femtosecond optical pulses in an X-ray free electron laser. *Journal of Applied Physics* **2017**, *122*, 203105. doi:10.1063/1.5012749.
12. Togashi, T.; Owada, S.; Kubota, Y.; Sueda, K.; Katayama, T.; Tomizawa, H.; Yabuuchi, T.; Tono, K.; Yabashi, M. Femtosecond Optical Laser System with Spatiotemporal Stabilization for Pump-Probe Experiments at SACLA. *Applied Sciences* **2020**, *10*, 7934. doi:10.3390/app10217934.
13. Greisman, J.B.; Dalton, K.M.; Hekstra, D.R. reciprocalspaceship : a Python library for crystallographic data analysis. *Journal of Applied Crystallography* **2021**, *54*, 1521–1529. doi:10.1107/S160057672100755X.
14. Terwilliger, T.C.; Berendzen, J. Bayesian Difference Refinement. *Acta Crystallographica Section D Biological Crystallography* **1996**, *52*, 1004–1011. doi:10.1107/S0907444996006725.
15. Ursby, T.; Bourgeois, D. Improved Estimation of Structure-Factor Difference Amplitudesfrom Poorly Accurate Data. *Acta Crystallographica Section A Foundations of Crystallography* **1997**, *53*, 564–575. doi:10.1107/S0108767397004522.
16. Winn, M.D.; Ballard, C.C.; Cowtan, K.D.; Dodson, E.J.; Emsley, P.; Evans, P.R.; Keegan, R.M.; Krissinel, E.B.; Leslie, A.G.W.; McCoy, A.; McNicholas, S.J.; Murshudov, G.N.; Pannu, N.S.; Potterton, E.A.; Powell, H.R.; Read, R.J.; Vagin, A.; Wilson, K.S. Overview of the CCP 4 suite and current developments. *Acta Crystallographica Section D Biological Crystallography* **2011**, *67*, 235–242. doi:10.1107/S0907444910045749.
17. Ren, Z.; Perman, B.; Šrajer, V.; Teng, T.Y.; Pradervand, C.; Bourgeois, D.; Schotte, F.; Ursby, T.; Kort, R.; Wulff, M.; Moffat, K. A Molecular Movie at 1.8 Å Resolution Displays the Photocycle of Photoactive Yellow Protein,

a Eubacterial Blue-Light Receptor, from Nanoseconds to Seconds †. *Biochemistry* **2001**, *40*, 13788–13801. doi:10.1021/bi0107142.

18. Schmidt, M.; Rajagopal, S.; Ren, Z.; Moffat yz, K. Application of Singular Value Decomposition to the Analysis of Time-Resolved Macromolecular X-Ray Data. *Biophysical Journal* **2002**, *84*, 2112–2129.
19. Pandey, S.; Bean, R.; Sato, T.; Poudyal, I.; Bielecki, J.; Cruz Villarreal, J.; Yefanov, O.; Mariani, V.; White, T.A.; Kupitz, C.; Hunter, M.; Abdellatif, M.H.; Bajt, S.; Bondar, V.; Echelmeier, A.; Doppler, D.; Emons, M.; Frank, M.; Fromme, R.; Gevorkov, Y.; Giovanetti, G.; Jiang, M.; Kim, D.; Kim, Y.; Kirkwood, H.; Klimovskaia, A.; Knoska, J.; Koua, F.H.M.; Letrun, R.; Lisova, S.; Maia, L.; Mazalova, V.; Meza, D.; Michelat, T.; Ourmazd, A.; Palmer, G.; Ramilli, M.; Schubert, R.; Schwander, P.; Silenzi, A.; Sztuk-Dambietz, J.; Tolstikova, A.; Chapman, H.N.; Ros, A.; Barty, A.; Fromme, P.; Mancuso, A.P.; Schmidt, M. Time-resolved serial femtosecond crystallography at the European XFEL. *Nature Methods* **2020**, *17*, 73–78. doi:10.1038/s41592-019-0628-z.
20. Murshudov, G.N.; Skubák, P.; Lebedev, A.A.; Pannu, N.S.; Steiner, R.A.; Nicholls, R.A.; Winn, M.D.; Long, F.; Vagin, A.A. REFMAC5 for the refinement of macromolecular crystal structures. *Acta crystallographica. Section D, Biological crystallography* **2011**, *67*, 355–67. doi:10.1107/S0907444911001314.
21. Kabsch, W. XDS. *Acta crystallographica. Section D, Biological crystallography* **2010**, *66*, 125–32. doi:10.1107/S0907444909047337.
22. Pronk, S.; Páll, S.; Schulz, R.; Larsson, P.; Bjelkmar, P.; Apostolov, R.; Shirts, M.R.; Smith, J.C.; Kasson, P.M.; van der Spoel, D.; Hess, B.; Lindahl, E. GROMACS 4.5: a high-throughput and highly parallel open source molecular simulation toolkit. *Bioinformatics* **2013**, *29*, 845–854. doi:10.1093/bioinformatics/btt055.
23. Morozov, D. Modified GROMACS 4.6.5 **2022**. doi:10.5281/ZENODO.6669552.
24. Titov, A.V.; Ufimtsev, I.S.; Luehr, N.; Martinez, T.J. Generating Efficient Quantum Chemistry Codes for Novel Architectures. *Journal of Chemical Theory and Computation* **2013**, *9*, 213–221. doi:10.1021/ct300321a.
25. Ufimtsev, I.S.; Martinez, T.J. Quantum Chemistry on Graphical Processing Units. 3. Analytical Energy Gradients, Geometry Optimization, and First Principles Molecular Dynamics. *Journal of Chemical Theory and Computation* **2009**, *5*, 2619–2628. doi:10.1021/ct9003004.
26. Barca, G.M.J.; Bertoni, C.; Carrington, L.; Datta, D.; De Silva, N.; Deustua, J.E.; Fedorov, D.G.; Gour, J.R.; Gunina, A.O.; Guidez, E.; Harville, T.; Irle, S.; Ivanic, J.; Kowalski, K.; Leang, S.S.; Li, H.; Li, W.; Lutz, J.J.; Magoulas, I.; Mato, J.; Mironov, V.; Nakata, H.; Pham, B.Q.; Piecuch, P.; Poole, D.; Pruitt, S.R.; Rendell, A.P.; Roskop, L.B.; Ruedenberg, K.; Sattasathuchana, T.; Schmidt, M.W.; Shen, J.; Slipchenko, L.; Sosonkina, M.; Sundriyal, V.; Tiwari, A.; Galvez Vallejo, J.L.; Westheimer, B.; Włoch, M.; Xu, P.; Zahariev, F.; Gordon, M.S. Recent developments in the general atomic and molecular electronic structure system. *The Journal of Chemical Physics* **2020**, *152*, 154102. doi:10.1063/5.0005188.
27. Granovsky, A.A. Extended multi-configuration quasi-degenerate perturbation theory: The new approach to multi-state multi-reference perturbation theory. *The Journal of Chemical Physics* **2011**, *134*, 214113. doi:10.1063/1.3596699.
28. Dunning, T.H. Gaussian basis sets for use in correlated molecular calculations. I. The atoms boron through neon and hydrogen. *The Journal of Chemical Physics* **1989**, *90*, 1007–1023. doi:10.1063/1.456153.
29. Duan, Y.; Wu, C.; Chowdhury, S.; Lee, M.C.; Xiong, G.; Zhang, W.; Yang, R.; Cieplak, P.; Luo, R.; Lee, T.; Caldwell, J.; Wang, J.; Kollman, P. A point-charge force field for molecular mechanics simulations of proteins based on condensed-phase quantum mechanical calculations. *Journal of Computational Chemistry* **2003**, *24*, 1999–2012. doi:10.1002/jcc.10349.
30. Adamo, C.; Barone, V. Toward reliable density functional methods without adjustable parameters: The PBE0 model. *The Journal of Chemical Physics* **1999**, *110*, 6158–6170. doi:10.1063/1.478522.
31. Grimme, S.; Antony, J.; Ehrlich, S.; Krieg, H. A consistent and accurate ab initio parametrization of density functional dispersion correction (DFT-D) for the 94 elements H-Pu. *The Journal of Chemical Physics* **2010**, *132*, 154104. doi:10.1063/1.3382344.
32. Siegbahn, P.E.M.; Almlöf, J.; Heiberg, A.; Roos, B.O. The complete active space SCF (CASSCF) method in a Newton–Raphson formulation with application to the HNO molecule. *The Journal of Chemical Physics* **1981**, *74*, 2384–2396. doi:10.1063/1.441359.
33. Levine, B.G.; Coe, J.D.; Martínez, T.J. Optimizing Conical Intersections without Derivative Coupling Vectors: Application to Multistate Multireference Second-Order Perturbation Theory (MS-CASPT2). *The Journal of Physical Chemistry B* **2008**, *112*, 405–413. doi:10.1021/jp0761618.

34. Polyakov, I.V.; Grigorenko, B.L.; Epifanovsky, E.M.; Krylov, A.I.; Nemukhin, A.V. Potential Energy Landscape of the Electronic States of the GFP Chromophore in Different Protonation Forms: Electronic Transition Energies and Conical Intersections. *Journal of Chemical Theory and Computation* **2010**, *6*, 2377–2387. doi:10.1021/ct100227k.
35. Weber, W.; Helms, V.; McCammon, J.A.; Langhoff, P.W. Shedding light on the dark and weakly fluorescent states of green fluorescent proteins. *Proceedings of the National Academy of Sciences* **1999**, *96*, 6177–6182. doi:10.1073/pnas.96.11.6177.
36. El Khatib, M.; Martins, A.; Bourgeois, D.; Colletier, J.P.; Adam, V. Rational design of ultrastable and reversibly photoswitchable fluorescent proteins for super-resolution imaging of the bacterial periplasm. *Scientific Reports* **2016**, *6*, 18459. doi:10.1038/srep18459.
37. Wagner, A.; Duman, R.; Henderson, K.; Mykhaylyk, V. In-vacuum long-wavelength macromolecular crystallography. *Acta Crystallographica Section D Structural Biology* **2016**, *72*, 430–439. doi:10.1107/S2059798316001078.
38. Thorn, A.; Sheldrick, G.M. ANODE : anomalous and heavy-atom density calculation. *Journal of Applied Crystallography* **2011**, *44*, 1285–1287. doi:10.1107/S0021889811041768.
39. Adam, V.; Hadjidemetriou, K.; Jensen, N.; Shoeman, R.L.; Woodhouse, J.; Aquila, A.; Banneville, A.S.; Barends, T.R.M.; Bezchastnov, V.; Boutet, S.; Byrdin, M.; Cammarata, M.; Carbajo, S.; Christou, N.E.; Coquelle, N.; Mora, E.D.l.; Khatib, M.E.; Chicano, T.M.; Doak, R.B.; Fieschi, F.; Foucar, L.; Glushonkov, O.; Gorel, A.; Grünbein, M.L.; Hilpert, M.; Hunter, M.; Kloos, M.; Koglin, J.E.; Lane, T.J.; Liang, M.; Mantovanelli, A.; Nass, K.; Kovacs, G.N.; Owada, S.; Roome, C.M.; Schirò, G.; Seaberg, M.; Stricker, M.; Thépaut, M.; Tono, K.; Ueda, K.; Uriarte, L.M.; You, D.; Zala, N.; Domratcheva, T.; Jakobs, S.; Sliwa, M.; Schlichting, I.; Colletier, J.P.; Bourgeois, D.; Weik, M. Rational control of structural off-state heterogeneity in a photoswitchable fluorescent protein provides switching contrast enhancement. *bioRxiv* **2021**, p. 2021.11.05.462999. doi:10.1101/2021.11.05.462999.
